# Heat wave event facilitates defensive responses in invasive C3 plant *Ambrosia artemisiifolia* L. under elevated CO_2_ concentration to the detriment of *Ophraella communa*

**DOI:** 10.3389/fpls.2022.907764

**Published:** 2022-07-27

**Authors:** Zhenya Tian, Chao Ma, Chenchen Zhao, Yan Zhang, Xuyuan Gao, Zhenqi Tian, Hongsong Chen, Jianying Guo, Zhongshi Zhou

**Affiliations:** ^1^State Key Laboratory for Biology of Plant Diseases and Insect Pests, Institute of Plant Protection, Chinese Academy of Agricultural Sciences, Beijing, China; ^2^National Nanfan Research Institute, Chinese Academy of Agricultural Sciences, Sanya, China; ^3^College of Plant Protection, Henan Agricultural University, Zhengzhou, China; ^4^Guangxi Key Laboratory for Biology of Crop Diseases and Insect Pests, Institute of Plant Protection, Guangxi Academy of Agricultural Sciences, Nanning, China

**Keywords:** common ragweed, invasive plant, biological invasions, climate change, secondary metabolite, herbivore, *Ophraella communa*

## Abstract

To predict and mitigate the effects of climate change on communities and ecosystems, the joint effects of extreme climatic events on species interactions need to be understood. Using the common ragweed (*Ambrosia artemisiifolia* L.)—leaf beetle (*Ophraella communa*) system, we investigated the effects of heat wave and elevated CO_2_ on common ragweed growth, secondary metabolism, and the consequent impacts on the beetle. The results showed that elevated CO_2_ and heat wave facilitated *A. artemisiifolia* growth; further, *A. artemisiifolia* accumulated large amounts of defensive secondary metabolites. Being fed on *A. artemisiifolia* grown under elevated CO_2_ and heat wave conditions resulted in the poor performance of *O. communa* (high mortality, long development period, and low reproduction). Overall, under elevated CO_2_, heat wave improved the defensive ability of *A. artemisiifolia* against herbivores. On the other hand, enhanced adaptability to climatic changes may aggravate invasive plant distribution, posing a challenge to the control of invasive plants in the future.

## Abstract (key points)

Key message

- Being fed on *A. artemisiifolia* grown under elevated CO_2_ and heat wave conditions resulted in the poor performance of *O. communa* (high mortality, long development period, and low reproduction).
- Elevated CO_2_ and heat waves resulted in the accumulation of secondary metabolites in *A. artemisiifolia*.
- After feeding on the secondary metabolites accumulated by *A. artemisiifolia*, the mortality of *O. communa* increased.

## Introduction

Global warming is one of the hottest issues and has the potential to dramatically change environments worldwide (Vinagre et al., 2018). It is known that climate change is likely to impact species both directly and indirectly (Drake et al., 2017; Tong et al., 2017; Sun et al., 2018). Heat wave events are a universal climatic phenomenon in meteorology and occur when the daily maximum temperature of more than 5 consecutive days exceeds the average maximum temperature by 5°C (García-Herrera et al., 2010). In recent years, heat waves have imposed severe stresses on natural and human systems (Skinner et al., 2018; Seo et al., 2019). Heat waves can increase crop failure and the loss of livestock (García-Herrera et al., 2010) and reduce the gross primary productivity of vegetation (Ciais et al., 2005). Heat waves will increase in intensity, duration, and frequency because of global climate change (Seo et al., 2019). On the other hand, increasing CO_2_ is also a common phenomenon in global climate change (Sun et al., 2018). In 2016, the CO_2_ concentration was 46% higher than that in pre-industrial times (WMO Greenhouse Gas Bulletin, 2018), and it is predicted to reach 750 ppm by the end of the century (IPCC, 2013).

Elevated CO_2_ is known to alter an array of plant traits, and the typical effects of elevated CO_2_ on plants include increases in the photosynthetic rate, biomass, and carbon: nitrogen (C: N) ratio and affect the production of plant secondary metabolites, especially in C_3_ plants (Körner, 2000; Chen et al., 2005; Ainsworth and Rogers, 2007; Stiling and Tatiana, 2007). Many previous studies have demonstrated that, under elevated CO_2_ conditions, secondary defensive substances increase in plants, thus indirectly affecting the development and reproduction of herbivorous insects (Lincoln et al., 1993; Poorter et al., 1997; Curtis and Wang, 1998; Schädler et al., 2007). Especially even more dramatic reductions in the larval performance of herbivorous insects have been observed in some herbaceous C3 plants under elevated CO_2_ conditions (Karowe, 2007). Previous studies have revealed that mortality over the entire larval period increased by 180% for buckeye larvae feeding on plantain grown at 700 ppm CO_2_ condition (Fajer, 1989) and by 135% for leaf miners feeding on Paterson’s Curse at 700 ppm CO_2_ condition (Johns and Hughes, 2002).

It is known that higher temperatures are always accompanied by elevated atmospheric CO_2_ concentrations (Lee et al., 2020), and the global mean surface temperature is expected to rise by 2.5–4°C, depending on the scenario, by the year 2100 (IPCC, 2021). Few case studies have explored the effects of elevated CO_2_ and elevated temperature on herbivore performance in such a way that both individual and combined effects of these two factors could be evaluated (Zvereva and Kozlov, 2006). Several studies have concluded that temperature does not affect plant and herbivorous insect responses to CO_2_ elevation (Buse et al., 1998; Williams et al., 2000), while others have demonstrated strong interactive effects of CO_2_ and temperature (Johns and Hughes, 2002; Veteli et al., 2002; Johns et al., 2003). However, a more recent study has demonstrated that relatively low concentrations of plant secondary metabolites were observed when plants were exposed to the combined impact of elevated CO_2_ and temperature, and the responses of both phenolics and terpenes to CO_2_ were strongly modified by elevated temperatures. Therefore, elevated temperatures can lead to the amelioration of the negative effects of CO_2_ on herbivore fitness (Zvereva and Kozlov, 2006); in fact, elevated temperatures may counteract the defense-inducing effect of elevated CO_2_ on plants (Veteli et al., 2002, 2007; Zvereva and Kozlov, 2006). However, even when the total heat sum is the same, compared with a long-term increase in constant temperature, heat waves have more severe negative effects on plant physiology and communities (Bauweraerts et al., 2014).

Common ragweed, *Ambrosia artemisiifolia* L. (Asteraceae) is an invasive weed originating from North America. It is a C_3_ plant that often causes large losses to agriculture (Cowbrough et al., 2003). In addition, 13.5 million persons suffer from *Ambrosia*-induced allergies in Europe, causing costs of €7.4 billion annually (Schaffner et al., 2020). Some previous studies have revealed that the growth and reproduction of *A. artemisiifolia* were enhanced when grown under elevated CO_2_ conditions (Stinson and Bazzaz, 2006), and its pollen production was also dramatically increased at elevated CO_2_ levels (Ziska and Caulfield, 2000; Wayne et al., 2002; Rogers et al., 2006). Physiological and morphological mechanisms by which elevated CO_2_ enhances the status of this species as an agricultural pest and allergenic weed have been demonstrated (Stinson et al., 2006). Increased levels of individual flavonoid metabolites in *A. artemisiifolia* were found under elevated CO_2_ conditions (El Kelish et al., 2014). Currently, however, there are no reports on the impact of climate change on common ragweed’s natural herbivorous enemies, which is crucial for the prediction of common ragweed–herbivorous interactions in the future, and it may also be important for the prediction of biological control of *A. artemisiifolia* through *O. communa* herbivore.

In this study, we first examined whether the secondary metabolites of the C_3_ plant *A. artemisiifolia* increase when the plant experiences heat wave events at elevated CO_2_ levels. Second, we explored whether secondary metabolic changes in *A. artemisiifolia* have adverse effects on the performance of the specialist herbivorous insect *O. communa*. Based on the results of this study, we will be able to predict whether exposure to heat wave events under elevated CO_2_ conditions in the future will facilitate the defensive ability of these invasive C_3_ plants to withstand insect herbivores more effectively. In addition, we will be able to predict whether heat wave events will relieve harm of *A. artemisiifolia* to human under elevated CO_2_ in the future.

## Materials and methods

### Part 1: Effects of high CO_2_ concentration and heat wave on the performance of *Ambrosia artemisiifolia* and *Ophraella communa*

#### Control of CO_2_ concentrations

Experiments were conducted in four climate-controlled chambers (PRX-450C-CO_2_; Ningbo Haishu Saifu Experimental Instrument Factory, Ningbo, China) located at the Langfang Experimental Farm of Hebei Province, China (E 116°36′46″, N 39°30′36″, at an altitude of 27 m). Growth chambers were used rather than open-top chambers or Free-Air CO_2_ Enrichment (FACE), to provide stable CO_2_, light, and humidity for continuous treatment effect throughout the experimental period. The CO_2_ concentrations were set at the low atmospheric CO_2_ concentration (370 ppm) and at an elevated CO_2_ concentration (700 ppm, which is predicted for the end of this century; IPCC, 2013). Two blocks were used for each of the two CO_2_ treatments, and each block contained one pair of climate-controlled chambers (one with ambient CO_2_ and one with elevated CO_2_; Sun et al., 2018). The CO_2_ concentration in each chamber was maintained by continual flushing with CO_2_-free air and then re-injecting CO_2_ from the cylinders to maintain the appropriate CO_2_ concentration. CO_2_ concentrations in all chambers were monitored using an absolute infrared gas analyzer (MSA Instruments, Pittsburgh, PA, United States).

#### Host plants

*Ambrosia artemisiifolia* seeds used in this study were collected from the Langfang Experimental Farm in the last third of October in 2014. The seeds were then stored in a freezer at 4°C. Adequately stored seeds were germinated in a greenhouse [temperatures was 28°C; a 70 ± 5% relative humidity (RH); and photoperiod was 14/10 h (light/dark)] in late April 2015. When the seedlings reached a height of approximately 10 cm, seedlings were thinned to one per pot (21 cm diameter and 17 cm height) in all treatments filled with vermiculite, nutrient soil (It is made up of fertile field soil and decomposed fertilizer), and natural soil (Fine soil collected in the field) in the volumetric ratio of 1:2:3. As experiments only examined the seedlings over 10 weeks, the plastic pot size was not a limiting factor. Each pot was fertilized weekly with a commercial fertilizer (Stanley Agriculture Group Co., Ltd., Linyi, China) and irrigated with 1 L of water every 3 days. For the experiment, 40 potted plants were evenly spaced in four climate-controlled chambers, two with 370 ppm and the other two with 700 ppm CO_2_ concentration, respectively. Apart from the differing CO_2_ concentrations, the growth temperature conditions for the *A. artemisiifolia* seedlings before heat wave stresses were as follows: day/night temperatures were maintained at 30/25°C for 14/10 h; a 70 ± 5% relative humidity (RH) and a mean photosynthetic photon flux density (PPFD) of 600 μmol photons m^−2^ s^−1^ were maintained at all time. To avoid position effects, pots were rotated randomly inside each chamber (length = 80 cm, width = 75 cm, and height = 185 cm) and moved from one chamber to the other every 5 days, maintaining the corresponding CO_2_ treatment throughout the experiment.

#### Heat wave treatment

At 70 days after sowing, ragweed seedlings were subjected to a heat wave stress treatment. To investigate the profound effects of heat wave on *A. artemisiifolia–O. communa* interactions, the time of heat wave treatment was 5 days in this study based on the definition of heat wave (García-Herrera et al., 2010). Half of each group of seedlings in the two CO_2_ concentration chambers described above were selected randomly for further cultivation under the original conditions, while the other half were transferred to two other growth chambers (one with 370 ppm and the other with 700 ppm of CO_2_) to undergo heat wave treatment. Therefore, there were 10 potted seedlings in each of the four growth chambers, which were provided the following treatment conditions: (1) ambient atmosphere CO_2_ concentration and ambient temperature (370 ppm, 30/25°C for 14/10 h in 1 day), acting as the control (AC + AT); (2) ambient atmosphere CO_2_ concentration and heat wave (370 ppm, 40°C for 8 h, and 30/25°C for 6/10 h in 1 day), acting as the heat wave treatment (AC + HW); (3) elevated atmospheric CO_2_ concentration and ambient temperature (700 ppm, 30/25°C for 14/10 h in 1 day), acting as the elevated CO_2_ treatment (EC + AT); (4) elevated atmospheric CO_2_ concentration and heat wave (700 ppm, 40°C for 8 h, and 30/25°C for 6/10 h in 1 day), acting as the combination treatment of elevated CO_2_ and heat wave (EC + HW). The heat wave stress was imposed by increasing the chamber temperature to 40°C during the daytime for 8 h daily for 5 days. Each day, plants received 8 h of heat wave, starting at 9:00 and ending at 17:00, and the rest of the 24 h period remained at the same temperature as that of the control treatment (i.e., day temperatures from 6:00 to 9:00 and 17:00 to 20:00 were 30°C, and night temperatures from 20:00 to 5:00 were 25°C). The plants were watered and fertilized the same as before. After the 5-day heat wave treatment, heat wave treatment chambers were returned to the same air temperature as that of the ambient treatment (30/25°C for 14/10 h). Methods in this part referred to Tripathee (2008).

#### Growth data collection

Plant height and CO_2_ assimilation were measured five times: at the end of day 0, 5, 10, 15, and 20 post-heat wave treatment. The total leaf area of plants was measured on day 20 after heat wave treatment. CO_2_ assimilation was measured on single, young, fully expanded leaves using a portable photosynthesis system (HCM-1000; Walz, Germany) equipped with a 6 cm^2^ leaf chamber. Measurements were made at ambient light intensity within 1 min of insertion of leaves into the environmentally controlled cuvette and after stabilization of CO_2_ flux, to ensure that photosynthetic response reflected conditions within the growth chambers. Total plant leaf area was determined using a portable leaf area meter (model CI-202, CID, Inc., Camas, WA, United States). Plant height was measured with a tape measure from the apex of the plant to the soil surface. Five plants were randomly selected for measurement per treatment.

#### Plant material sampling and foliar chemistry

Leaves from plants in the treatments were sampled at five times over the course of the experiment, and five replicate plants per treatment. Samples were collected at the same time as that when the plant measurements described above were undertaken. Sampled leaves (≥5 g) were randomly selected and, after being picked, were immediately frozen in liquid nitrogen and stored at −80°C. Before chemical analysis of leaf tissue, leaves were dried in a circulating air oven at 68°C for 48 h, ground in liquid nitrogen, and kept in plastic containers that were placed in a desiccator and stored in a cold room at 4°C until time of use.

Extraction and quantification of total phenolic concentration followed the methodology of Ibrahim and Jaafar (2012). Briefly, finely ground leaf tissues (0.25 mm fragments) of standard weight (0.1 g) were extracted with 80% ethanol (10 ml) in an orbital shaker for 120 min at 50°C. The mixture was then filtered (Whatman™ No. 1), and the filtrate was used for the quantification of total phenolics. Folin–Ciocalteu reagent (diluted 10-fold) was used to determine the total phenolic content of the leaf samples. Sample extract (200 μl, after filtration) was mixed with Folin–Ciocalteu reagent (1.5 ml) and allowed to stand at 22°C for 5 min before adding NaNO_3_ solution (1.5 ml, 60 g L^−1^). After 2 h at 22°C, the absorbance at 725 nm was measured. The results were expressed as mg g^−1^ gallic acid equivalent (mg GAE g^−1^ dry sample).

Concentrations of condensed tannin were measured using the acid butanol method of Kanerva and Smolander (2008). Approximately, 0.1 g of ground sample was extracted with 1 ml of ice-cold 70% acetone containing 10 mM ascorbic acid at 4°C for 30 min. After centrifugation, the supernatants were pooled. After diluting the pooled sample supernatants with 350 μl of ascorbic acid, a 150-μl aliquot was reacted with 3.0 ml of 19:1 (v/v) N-butanol: concentrated HCl and 100 μl of 0.02 g ml^−1^ FeNH_4_(SO_4_)2·12H_2_O in 2 N HCl and incubated in a water bath (at 100°C) for 50 min. The absorbance of the solution at 550 nm was measured to quantify the condensed tannins. The reported total phenolics and condensed tannin concentrations are the means of five replicates.

#### Insects

More than 1,000 *O. communa* adults were collected from the town of Dajing (28°56′26″N, 113°14′38″E) in Miluo County, Yueyang City, Hunan Province, China, on June 24, 2014. Colonies of the beetle were maintained on *A. artemisiifolia* plants in the laboratory at 27 ± 1°C at the Langfang Experimental Farm of Hebei Province, China. Pupae were collected and stored in a transparent plastic box (19 × 12 × 6 cm^3^) and kept in an air-conditioned laboratory at 28 ± 1°C. Newly emerged adults were collected, and males and females were separately held on potted *A. artemisiifolia* plants in cages (40 cm × 40 cm × 60 cm) in the same laboratory, at a density of 20 adults per plant and one plant per cage. Two-day-old adults were used for the experiments.

#### Development and survivorship of immature *Ophraella communa*

The development and survivorship of immature *O. communa* were measured following the methodology of Zhou et al. (2010). After *A. artemisiifolia* plants have undergone the above four treatments (AC + AT, AC + HW, EC + AT, and EC + HW, respectively), *O. communa* eggs were raised on *A. artemisiifolia* leaves until adult emergence. Then *O. communa* and *A. artemisiifolia* were placed in the environmental chamber with corresponding CO_2_ concentration. Next, adults in four treatments were paired, and raised by untreated *A. artemisiifolia* plants grown in greenhouse. Eggs were checked daily until all had hatched. Each treatment was repeated five times in the same environmental chamber, and the next cohort of eggs was placed when all larvae in the previous treatment became pupae. Survival rates and developmental periods at different developmental stages were recorded.

#### Longevity and fecundity of leaf beetle adults

Twigs of common ragweed were inserted into plastic bottles (3 cm × 5 cm, diameter × height) filled with water and with a 0.8-cm-diameter hole in the lid to hold the twig, twigs are extracted from untreated common ragweed grown in greenhouse. Newly emerged *O. communa* adults in the four treatments were mated, and each pair was placed on fresh common ragweed twigs. Twigs with beetles were placed in transparent plastic boxes (19 × 12 × 6 cm^3^) covered with a mesh to prevent beetles from escaping. These plastic boxes with *O. communa* adults and *A. artemisiifolia* twigs were placed in environmental chambers under the same conditions as their immature stages. Fresh twigs were changed daily, and the eggs of *O. communa* were counted. The observation ended when the female adult died. If the male died earlier, another newly emerged male was added. Pre-oviposition and oviposition periods, longevity, and number of eggs laid per female were recorded.

#### Determination of enzyme activity

Three-day *O. communa* adults in part “Development and survivorship of immature *O. communa*” were used to determine enzyme activity. Catalase (CAT) activity was determined by the method based on Aebi (1984). The activity of superoxide dismutase (SOD) was determined using the method of Marklund and Marklund (1974). Acetylcholinesterase (AChE) activity was determined using acetylcholinesterase iodide (atchl) as a substrate (Ellman et al., 1961). Carboxylesterase (CarE) activity was determined using the methodology of Kandil et al. (2017). According to the method of Visweshwar et al. (2015), Nα-benzoyl-L-arg-*p*-nitroanilide (Ba*p*NA; Sigma Aldrich) was used as the specific substrate to determine the activity of trypsin. Each enzyme test was repeated three times, with six insects in each repeat.

### Part 2: Metabonomic profile of common ragweed after high CO_2_ concentration and heat wave treatment

Based on the results of *A. artemisiifolia* foliar chemistry and *O. communa* life table, we found that the *A. artemisiifolia* foliar chemistry and *O. communa* life table data between EC + HW and AC + AT treatment had the most obvious difference, so we send common ragweed leaf samples at the end of the control treatment (AC + AT) and the combination treatment of elevated CO_2_ and heat wave (EC + HW) to analyze changes in all metabolites using metabolomics methods.

#### Leaf sampling and preparation

Leaves from plants in the treatments were sampled 5 days after the heat wave treatment. Sampled leaves were randomly selected and were immediately frozen in liquid nitrogen after being picked and stored at −80°C. Each treatment had three replicates.

The metabolomics methods were as described by Chen et al. (2013). The freeze-dried leaves were crushed (30 Hz, 1.5 min) to powder by a mixer mill (MM 400; Retsch). Then, 100 g of the leaf powder was dissolved in methanol (Chromatographic purity, Merck) extract. The solution was placed in a refrigerator at 4°C overnight, and the extraction rate was improved by vortexing three times. After centrifugation (rotation speed 10,000 × *g*, 10 min), the supernatant was aspirated, and the sample was filtered with a microporous membrane (0.22 μm pore size) and stored in the injection bottle for liquid chromatography (LC)-mass spectrometry (MS)/MS analysis.

Additionally, to ensure data quality for metabolic profiling, quality control (QC) samples were prepared—by pooling aliquots of all samples that were representative of those under analysis—and used for data normalization. QC samples were prepared and analyzed using the same procedure as that for the experimental samples in each batch.

#### Ultra-performance LC–MS/MS analysis

The metabolomics methods used were as described by Chen et al. (2013). The LC–MS/MS portion of the platform was based on a UHPLC system (1,290 series, Agilent Technologies) equipped with an ACQUITY UPLC BEH Amide column (1.7 μm, 2.1 mm × 100 mm, Wasters) and a triple quadruple mass spectrometer (5500 QTRAP, AB SCIEX) in the multiple reaction monitoring (MRM) mode. Metabolites were detected in electrospray negative-ionization and positive-ionization modes. Samples (2 μl) were injected sequentially. The Waters ACQUITY UPLC HSS T3 C18 (1.8 μm, 2.1 mm × 100 mm, Wasters) was heated to 40°C at a flow rate of 400 μl/min. The gradient was started with the solvent and water/acetonitrile ratio at 95/5% (V/V) for 11 min, after which the volume ratio was changed to 5/95% for 1 min over 11 min, and finally to 95/5% at 12.1 min, lasting 15 min.

### Part 3: Effects of different metabolites on *Ophraella communa*

Based on the results of metabolomics, we selected the significantly upregulated secondary metabolites of common ragweed exposed to heat waves and high concentrations of CO_2_. Then, we set up a bioassay experiment to detect the effect of upregulated secondary metabolites on leaf beetle survivorship.

#### Bioassay test

Pure products of the purchased substances were prepared into 2,000, 1,500, 1,000, and 500 ppm series multiples with acetone (analytical purity) for reserved use. The fresh common ragweed leaves were cut into disks with a diameter of approximately 1–2 cm. The leaf disks were put into the doubling solutions of three substances for 5 s and then removed. After the solvent evaporated, the leaf disks were placed in a Petri dish (diameter 9 cm) padded with filter paper. The bottom layer of the filter paper was put into a piece of absorbent cosmetic cotton to moisturize the leaf disks. After starvation treatment of the second instar larvae for 8 h, they were placed on the leaves in the Petri dish; 15 test larvae were treated, with four repetitions, and acetone was set up as a blank control. The Petri dish was placed in an incubator (day temperature of 30 ± 1°C, night temperature of 25 ± 1°C, RH of 70 ± 5%, illumination condition of 14:10 L:D, and light intensity of 30,000 lux). The larvae were fed for 5 days with leaves soaked in metabolite solution, which were set in four concentrations (500, 1,000, 1,500, and 2,000 ppm). After 5 days, the soaked common ragweed leaves were replaced with fresh leaves until pupation of the larvae. Fresh leaves were replaced every 24 h. The larvae were investigated, and their daily mortality in each period was recorded.

#### Statistical analysis

Data were checked for normality as appropriate (*p* > 0.05) and, if needed, were arcsine square-root or log-transformed. The data for growth, foliar chemistry, and the biological parameters of the *O. communa* adults fed on common ragweed under the four treatments were analyzed using a two-way ANOVA *via* IBM SPSS Statistics 20; the differences between means were analyzed by one-way ANOVA *via* IBM SPSS Statistics 20 (LSD test, P¼ 0.05; SPPS, Inc., Chicago, IL, United States).

Life table parameters were estimated using the TWO-SEX-MS Chart program (Chi and Smith, 2014) based on the age-stage and two-sex life table theory (Chi, 1988). The age-stage-specific survival rate (Sxj; x¼age, j¼stage), age-stage-specific fecundity (fxj), age-specific survival rate (lx), age-specific fecundity (mx), and population parameters (*r*, the intrinsic rate of increase; *k*, the finite rate of increase; *R*_0_, the net reproductive rate) were analyzed, all the data from five replications are integrated into one life table.

For the metabolomics data, the SIMCAP 14 software (Umetrics, Umeå, Sweden) was used for all multivariate data analyses and modeling. Data were mean-centered using Pareto scaling. Models were built on principal component analysis (PCA). The discriminating metabolites were obtained using a statistically significant threshold of fold change (FC) and two-tailed Student’s *t*-test (*p* value) on the normalized raw data. The *p* value was calculated by one-way ANOVA for multiple groups. Metabolites with FC greater than 1.5 and *p* value less than 0.05 were considered statistically significant metabolites. FC was calculated as the logarithm of the average mass response (area) ratio between two arbitrary classes. The identified differential metabolites were used to perform cluster analyses using the R package (cluster, factoextra).

The local database was used to analyze the metabolite information, and quantitative information was determined in the MRM mode. The identified substances were analyzed by Kyoto Encyclopedia of Genes and Genomes (KEGG), and the differences among different samples were analyzed by PCA and Orthogonal partial least squares discriminant analysis (OPLS-DA).

## Results

### Effects of high CO_2_ concentration and heat wave on the performance and foliar chemistry of common ragweed

In both temperature treatments, leaf photosynthesis in *A. artemisiifolia* under elevated CO_2_ was significantly higher than that under ambient CO_2_ during this study (first measurement: *F*_3,36_ = 219.32, *p* < 0.0001; second: *F*_3,36_ = 162.97, *p* < 0.0001; third: *F*_3,36_ = 122.49, *p* < 0.0001; fourth: *F*_3,36_ = 189.04, *p* < 0.0001; and fifth: *F*_3,36_ = 160.19, *p* < 0.0001). Under elevated CO_2_, leaf photosynthesis at the ambient air temperature was significantly higher than that in the heat wave treatment in all measurements except for the fourth measurement time period (day 15 post-heat stress). Meanwhile, there was no significant difference in leaf photosynthesis rate between ambient temperature and heat wave treatments under ambient CO_2_ conditions (*p* > 0.05), except for the fifth measurement, i.e., on day 20 post-heat stress (Supplementary Figure 1).

The total leaf area of *A. artemisiifolia* under elevated CO_2_ was significantly higher than that under ambient CO_2_ in both the temperature treatments (ambient and heat wave; *F*_3,16_ = 5.04, *p* = 0.012; Supplementary Figure 2). Under elevated CO_2_conditions, the total leaf area of *A. artemisiifolia* was significantly increased in the ambient temperature treatment compared with the heat wave stress treatment (*p* < 0.05).

At all five post-heat stress measurement periods, the heights of *A. artemisiifolia* under elevated CO_2_ were significantly greater than those under ambient CO_2_ (first measurement: *F*_3,16_ = 7.39, *p* = 0.0025; second: *F*_3,16_ = 5.29, *p* < 0.0100; third: *F*_3,16_ = 5.15, *p* < 0.0111; fourth: *F*_3,16_ = 3.74, *p* < 0.0329; and fifth: *F*_3,16_ = 4.82, *p* < 0.0141; Supplementary Figure 3). However, there was no significant difference in plant height between ambient temperature and the heat wave stress treatment under either ambient or elevated CO_2_ (*p* > 0.05).

**Figure 1:**
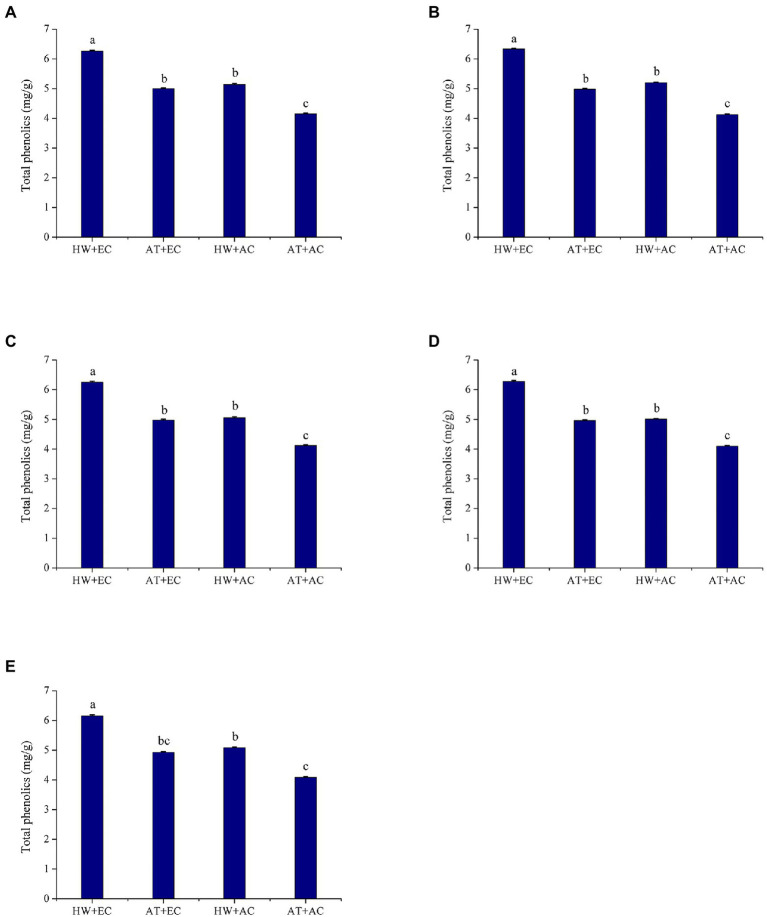
Total phenolic concentration (±SE) of *Ambrosia artemisiifolia* under different stress conditions (*n* = 5). Data represented by columns bearing the same letters were not significantly different (LSD, *p* = 0.05). **(A–E)** Represent the first, second, third, fourth, and fifth measurements, respectively. AT denotes the ambient temperature condition; HW denotes the heat wave condition. EC and AC represent elevated atmosphere CO_2_ concentration and ambient atmosphere CO_2_ concentration, respectively.

The combined treatment of elevated atmospheric CO_2_ concentration and heat wave (EC + HW) had a strong effect on the total phenolic contents in *A. artemisiifolia* leaves. Throughout the experiment, the highest concentration of total foliar phenolics was found in the EC + HW treatment, followed by the heat wave treatment (AC + HW); the lowest total phenolics occurred under the ambient atmospheric CO_2_ concentration and ambient temperature treatment (AC + AT; Figure 1). At all five sampling times, *A. artemisiifolia* treated with EC + HW had a significantly higher total phenolic concentration than that in *A. artemisiifolia* leaves in the other three treatments. Except at the third sampling time, heat wave remarkably increased the total phenolic concentration at both concentrations of CO_2_ (elevated and ambient). In both the ambient temperature and heat wave treatments, elevated atmospheric CO_2_ concentration also increased total phenolics concentration on the first, second, and fourth sampling times (first: *p* = 0.001; second: *p* = 0.000; third: *p* = 0.002; fourth: *p* = 0.001; and fifth: *p* = 0.002).

**Figure 2:**
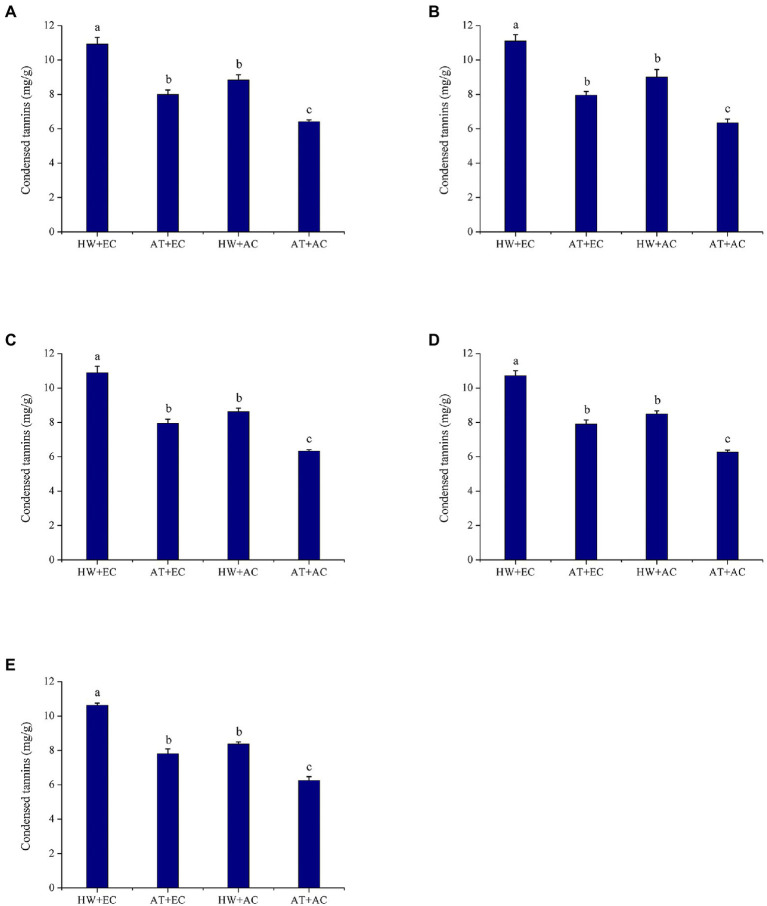
Condensed tannins concentration (±SE) of *Ambrosia artemisiifolia* under different stress conditions (*n* = 5). Data represented by columns bearing the same letters were not significantly different (LSD, *p* = 0.05). **(A–E)** Represent the first, second, third, fourth, and fifth measurements, respectively. AT denotes the ambient temperature condition; HW denotes the heat wave condition. EC and AC represent elevated atmosphere CO_2_ concentration and ambient atmosphere CO_2_ concentration, respectively.

Compared with the untreated control (AC + AT), elevated atmospheric CO_2_ concentration (EC + AT), heat wave (AC + HW), and their combination (EC + HW) significantly increased condensed tannin concentration in leaves at all five sampling times. Foliar condensed tannin concentrations were the highest in the EC + HW treatment, followed by the AC + HW treatment and finally the AC + AT treatment. Concentrations of condensed tannins were the lowest in AC + AT at all five sampling times (Figure 2). Regardless of CO_2_ concentration (or temperature), heat wave (or elevated atmospheric CO_2_ concentration) had a strongly positive effect on total tannin concentration in *A. artemisiifolia* leaves throughout the experiment (first: *p* < 0.001; second: *p* < 0.001; third: *p* < 0.001; fourth: *p* < 0.001; and fifth: *p* < 0.001).

### Effect on the development period, female ratio, adult longevity, and female fecundity of *Ophraella communa* after feeding on common ragweed grown under different treatment conditions

The development period of each stage, female ratio, adult longevity, and female fecundity of *O. communa* are presented in Tables 1, 2. The first larval stage, the whole larval stage, the pupal stage, and the entire immature stage of *O. communa* were significantly increased when beetles fed on common ragweed grown under the high CO_2_ concentration treatment. Furthermore, among the four treatments, *O. communa* that fed on common ragweed treated with a combination of high CO_2_concentration and heat wave (EC + HW) had the longest developmental period (*p* < 0.05). There was no significant difference in terms of the female ratio among *O. communa* populations fed on common ragweed grown under the four treatments. However, compared with the AC + AT treatment, the longevity of *O. communa* adults and the fecundity of females that fed on common ragweed treated with high CO_2_ concentration were significantly decreased (*p* < 0.05). The longevity of the *O. communa* adults that fed on common ragweed treated under EC + HW conditions was the shortest, and the difference in female longevity between the EC + AT and EC + HW treatments was significant. For fecundity, irrespective of the treatment of heat wave alone (AC + HW), high concentration CO_2_ alone (EC + AT), and a combination of high concentration CO_2_ and heat waves (EC + HW), the number of eggs laid per female was significantly decreased compared with the control (AC + AT) treatment (*p* < 0.05).

**Table 1:** Effect of different stages of *Ophraella communa* by feeding on common ragweed after treatment on development period.

Treatment	Developmental period (*d*)^a^						
	Egg	First instar larva	Second instar larva	Third instar larva	Larva stage^b^	Pupa	Entire immature^c^
AC + AT	5.1 ± 0.2 a	3.0 ± 0.0 b	2.7 ± 0.1 a	3.3 ± 0.2 a	9.1 ± 0.1 c	5.5 ± 0.1 d	19.8 ± 0.2 c
AC + HW	5.1 ± 0.1 a	3.3 ± 0.1 b	2.8 ± 0.1 a	3.6 ± 0.1 a	9.6 ± 0.2 bc	6.1 ± 0.0 c	20.8 ± 0.3 b
EC + AT	5.1 ± 0.1 a	3.7 ± 0.1 a	2.8 ± 0.1 a	3.7 ± 0.2 a	10.1 ± 0.3 ab	6.4 ± 0.1 b	21.7 ± 0.2 a
EC + HW	5.2 ± 0.1 a	3.9 ± 0.1 a	2.8 ± 0.1 a	3.7 ± 0.1 a	10.4 ± 0.2 a	6.7 ± 0.1 a	22.3 ± 0.3 a
*F*_3,16_	0.08	17.25	0.19	1.17	9.04	43.05	19.09
*p*	0.9708	<0.0001	0.9029	0.3507	0.0010	<0.0001	<0.0001

a Mean ± SE. Means within the same column followed by different letters are significantly different at *p* ≤ 0.05, according to ANOVA: LSD test. The same for the following tables.

b The larval stage refers to the developmental period of *O. communa* from first instar larva to pupa emergence. The same for the following tables.

c Entire immature refers to the developmental period of *O. communa* from egg to adult emergence. The same for the following table.

The lowercase letters after the numbers are used to show the significance of the differences between the data.

**Table 2:** Effect on female ratio, longevity of adult, and fecundity of female *Ophraella communa* after feeding on common ragweed grown under different treatment conditions.

Treatment	Female ratio (%)	Female longevity (*d*)	Male longevity (*d*)	No. eggs laid per female
AC + AT	53.7 ± 0.7 a	43.1 ± 0.6 a	56.6 ± 1.0 a	1137.0 ± 12.8 a
AC + HW	51.8 ± 3.1 a	42.6 ± 0.6 a	58.6 ± 0.3 a	1070.6 ± 7.6 b
EC + AT	50.8 ± 1.8 a	38.2 ± 0.7 b	53.7 ± 1.1 b	637.7 ± 33.4 c
EC + HW	52.0 ± 2.2 a	32.7 ± 1.2 c	52.5 ± 0.9 b	581.0 ± 23.9 c
*F*_3,16_	0.31	34.90	9.87	173.41
*p*	0.8152	<0.0001	0.0006	<0.0001

Means within the same column followed by different letters are significantly different at *p* ≤ 0.05 as determined by an ANOVA: LSD test.

The lowercase letters after the numbers are used to show the significance of the differences between the data.

### Effect on the survival rate of different stages of *Ophraella communa* after feeding on common ragweed grown under different treatments

As shown in Table 3, there was no significant difference in the survival rate of larvae when *O. communa* larvae were fed on common ragweed treated under the four stress conditions. However, the survival rate of pupa and the entire immature stage was significantly lower in *O. communa* fed on common ragweed treated under EC + HW than that of *O. communa* fed on common ragweed treated under AC + AT conditions.

**Table 3:** Effect on survival of *Ophraella communa* after feeding on common ragweed grown under different treatment conditions.

Treatment	Survival rate (%)						
	Egg	First instar larva	Second instar larva	Third instar larva	Larva stage^b^	Pupa	Entire immature^c^
AC + AT	85.8 ± 2.2a	75.3 ± 5.7a	92.6 ± 1.6a	92.9 ± 3.5a	64.1 ± 2.9a	97.1 ± 0.4a	53.3 ± 2.3a
AC + HW	84.4 ± 1.6a	73.6 ± 2.9a	92.1 ± 1.6a	92.0 ± 3.1a	61.2 ± 3.8a	94.4 ± 1.6ab	48.5 ± 2.8ab
EC + AT	82.4 ± 3.3a	81.7 ± 2.9a	90.0 ± 2.1a	90.2 ± 2.3a	66.5 ± 3.9a	87.6 ± 3.8b	46.8 ± 2.1ab
EC + HW	81.5 ± 2.3a	77.4 ± 5.8a	85.6 ± 5.4a	88.2 ± 4.7a	64.8 ± 5.1a	88.5 ± 3.7b	43.7 ± 3.4b
*F*_3,16_	0.62	0.59	1.06	0.36	0.30	2.79	2.19
*p*	0.6136	0.6287	0.3927	0.7850	0.8226	0.0745	0.1291

a Mean ± SE. Means within the same column followed by different letters are significantly different at *p* ≤ 0.05, according to ANOVA: LSD test. The same for the following tables.

b The larval stage refers to the developmental period of *O. communa* from first instar larva to pupa emergence. The same for the following tables.

c Entire immature refers to the developmental period of *O. communa* from egg to adult emergence.

### Effect on the life table parameters of *Ophraella communa* after feeding on common ragweed grown under different treatment conditions

Compared with the control group, the intrinsic rate of increase (*r*) and net reproduction rate (*R*o) decreased significantly and the generation time (T) increased significantly in *O. communa* fed on common ragweed grown under the other three treatments. There was no significant difference in the finite rate of increase (λ) among *O. communa* fed on common ragweed grown under the four treatments. In addition, *O. communa* fed on common ragweed treated under EC + HW conditions had the lowest values of *r*, *Ro*, and *λ*, and had the longest generation time, which were 0.1340, 129.4, 1.1435, and 36.2, respectively (Table 4).

**Table 4:** Effect on the life table parameters of *Ophraella communa* after feeding on common ragweed grown under different treatment conditions.

Treatment	Intrinsic rate of increase (*r*)	Net reproduction rate (*R*o)	Generation time (T)	Finite rate of increase (*λ*)
AC + AT	0.1821 ± 0.0022 a	324.6 ± 19.6 a	31.7 ± 0.4 c	1.3556 ± 0.1554 a
AC + HW	0.1698 ± 0.0018 b	265.4 ± 3.1 b	32.9 ± 0.3 b	1.1851 ± 0.0021 a
EC + AT	0.1511 ± 0.0019 c	159.9 ± 8.6 c	33.6 ± 0.4 b	1.1631 ± 0.0022 a
EC + HW	0.1340 ± 0.0026 d	129.4 ± 9.5 c	36.2 ± 0.3 a	1.1435 ± 0.0030 a
*F*_3,16_	98.48	59.09	28.00	1.57
*p*	<0.0001	<0.0001	<0.0001	0.2356

Mean ± SE. Means within the same column followed by different letters are significantly different at *p* ≤ 0.05, as determined by an ANOVA: LSD test.

The lowercase letters after the numbers are used to show the significance of the differences between the data.

**Figure 3:**
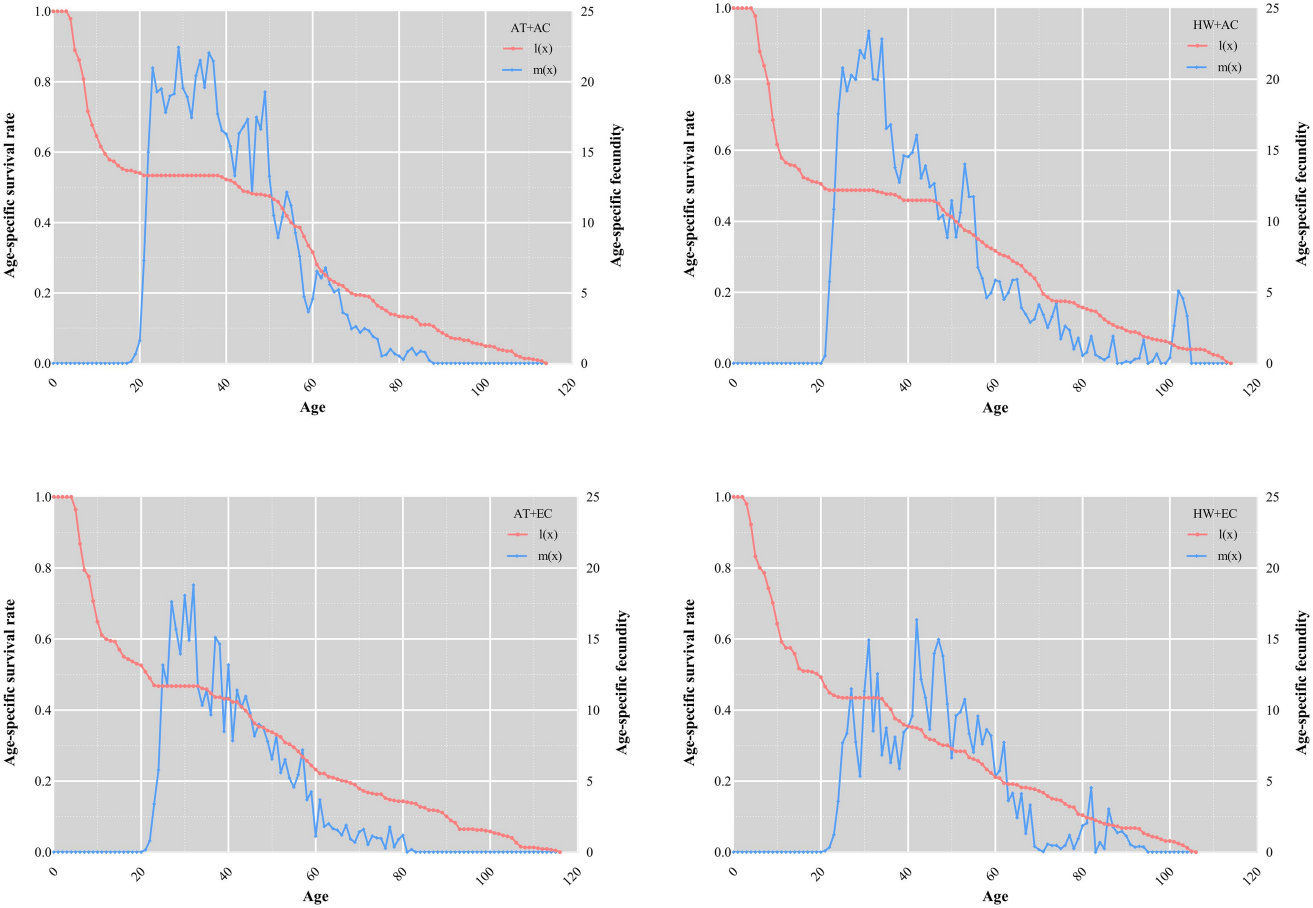
Effect on age-specific survivorship and fecundity of *Ophraella communa* after feeding on common ragweed grown under different treatment conditions. AT denotes the ambient temperature condition; HW denotes the heat wave condition. EC and AC represent elevated atmosphere CO_2_ concentration and ambient atmosphere CO_2_ concentration, respectively.

The maximum daily fecundity was 22.5, 23.4, 18.8, and 16.4 under AC + AT, AC + HW, EC + AT, and EC + HW, respectively; the peak fecundity occurred on days 29, 31, 32, and 42, respectively. The duration of the peak fecundity in the AC + AT treatment was longer and decreased gradually, while that of AC + HW and EC + AT treatments decreased rapidly after reaching the peak reproductive stage. Compared with the other three treatment groups, the peak reproductive stage of the EC + HW treatment group appeared later, decreased rapidly after reaching the peak, rose again, and then decreased gradually (Figure 1). The age-specific survivorship of each treatment decreased rapidly in 25 days, with the survivorship for AC + AT, AC + HW, EC + AT, and EC + HW decreasing rapidly after 39, 34, 34, and 34 days, respectively (Figure 3).

### Effect on enzyme activity of *Ophraella communa* after feeding on common ragweed grown under different treatment conditions

**Figure 4:**
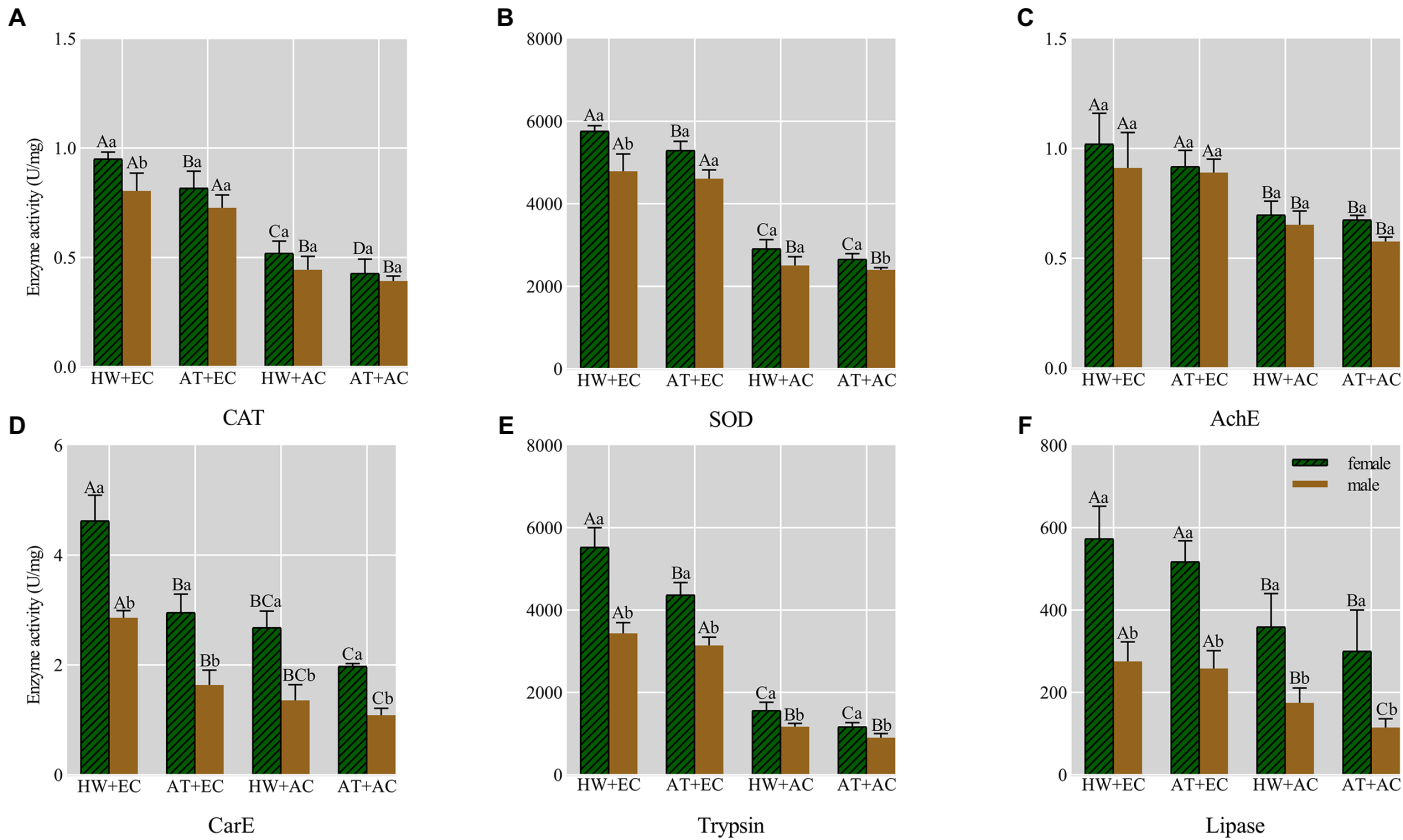
Effect on six enzyme activities of *Ophraella communa* after feeding on common ragweed grown under different treatment conditions. The green column denotes the female enzyme activity, the brown column denotes the male enzyme activity; AT denotes the ambient temperature condition; HW denotes the heat wave condition. EC and AC represent elevated atmosphere CO_2_ concentration and ambient atmosphere CO_2_ concentration, respectively; Different uppercase letters on the column indicate statistical differences between different genders, different lowercase letters on the column represent statistical differences between different treatment at *P* < 0.05, as determined by an ANOVA: LSD test.

Compared with common ragweed grown under AC + AT conditions, the enzyme activities (including four detoxification enzymes and two ingestion enzymes) of the adults were changed after feeding on ragweed grown under EC + HW, EC + AT, and AC + HW conditions; the enzyme activities of catalase (CAT), superoxide dismutase (SOD), acetylcholinesterase (AChE), carboxylesterase (CarE), tryphin, and lipase increased. In all experimental conditions, the enzyme activity of female adults was higher than that of males, and the different experimental conditions had no significant effect on this result. A comparison of the enzyme activities under the four experimental conditions showed that the changes in enzyme activities in the adults fed on common ragweed grown under EC + HW conditions were the most significant, having the greatest impact on enzyme activities (Figure 4).

### Metabonomic profile of common ragweed after high CO_2_ concentration and heat wave treatment

**Figure 5:**
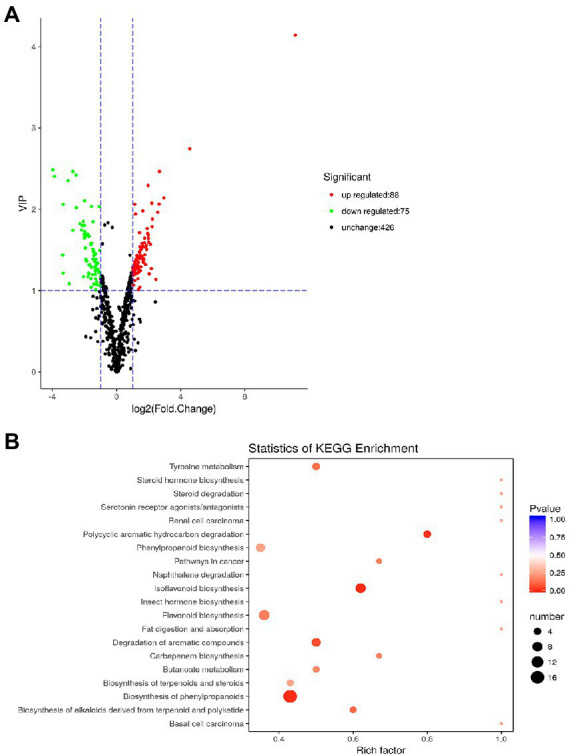
**(A)** Volcano plot of differentially expressed metabolites; **(B)** statistics of Kyoto Encyclopedia of Genes and Genomes (KEGG) enrichment. Leaves of plants under AC + AT and EC + HW conditions.

A total of 589 metabolites were measured from the leaves of plants under AC + AT and EC + HW conditions. Compared with the common ragweed growing in the AC + AT treatment, there were 163 different metabolites in the leaves of the common ragweed under the EC + HW treatment, 88 of which were significantly up regulated (VIP ≥ 1, FC ≥ 2) and 75 of which were significantly down regulated (VIP ≥ 1, FC ≤ 0.5, Figure 5A). In addition, the content of N-(p-coumaroyl) serotonin in common ragweed leaves under the EC + HW treatment was 2264.4-fold higher than that in the AC + AT treatment.

The classification results of KEGG annotation of different metabolites are shown in Supplementary Figure 4. There were163 different metabolites annotated in 116 different pathway species, including seven major pathway categories, most of which were concentrated in metabolism. Among them, 36 metabolites belonged to the metabolic pathway and 27 belonged to the biosynthesis of secondary metabolites, accounting for 54.55 and 40.91%, respectively. After enrichment of the KEGG pathway, it was found that the pathways of differential metabolites were mainly concentrated in flavonoid biosynthesis, isoflavonoid biosynthesis, and phenylpropanoid biosynthesis (Figure 5B).

### Bioassay test

Eight significantly upregulated substances [Cryptochlorogenic acid, N-(p-Cinnamoyl) serotonin, Glycitin, Phe-Phe, Fumaric acid, A-Ketoglutaric acid, Calycosin, and Glycitein] were used to perform the bioassay test. Compared with the control group, feeding on common ragweed leaves treated with cryptochlorogenic acid (2,000, 1,000, and 500 ppm), N-(p-Cinnamoyl) serotonin (2,000, 1,500, and 1,000 ppm), Glycitin (1,500 and 1,000 ppm), Phe-Phe (1,000 and 500 ppm), Fumaric acid (500 ppm), or A-Ketoglutaric acid (500 ppm) significantly increased the mortality of the leaf beetles (*p* < 0.05). In addition, the leaf beetles feeding on Calycosin- and Glycitein-treated leaves had higher mortality than the control group, although there was no significant difference (Table 5). For Glycitin, Phe-Phe, Fumaric acid, and A-Ketoglutaric acid, the trend of mortality showed a phenomenon of optimal lethal concentration, i.e., with the increase in the concentration of the substance provided, the mortality of the beetle increased gradually, but when higher concentration of substance was continually provided, mortality began to decrease.

**Table 5:** Effect on immature death rate of *Ophraella communa* after feeding on common ragweed grown under different treatment conditions.

Compounds	2,000 ppm (%)	1,500 ppm (%)	1,000 ppm (%)	500 ppm (%)
Cryptochlorogenic acid	51.7 ± 6.3*	41.7 ± 10.3	53.3 ± 4.7*	39.8 ± 3.6*
N-(p-Cinnamoyl)serotonin	42.6 ± 6.3*	48.3 ± 10.3*	45.0 ± 7.3*	41.7 ± 8.3
Glycitin	35.0 ± 11.0	45.0 ± 7.3*	36.7 ± 4.3*	35.5 ± 8.6
Phe-Phe	35.8 ± 6.3	33.3 ± 9.4	43.3 ± 8.3*	40.0 ± 2.7*
A-Ketoglutaric acid	25.0 ± 5.0	41.7 ± 9.9	28.3 ± 1.6	36.7 ± 1.9*
Fumaric acid	20.1 ± 9.3	30.0 ± 5.8	30.0 ± 4.3	43.3 ± 4.3*
Calycosin	31.7 ± 11.6	31.7 ± 4.2	31.7 ± 3.1	30.7 ± 4.8
Glycitein	28.3 ± 4.2	23.3 ± 4.3	28.3 ± 6.8	18.7 ± 1.8
Acetone	18.3 ± 5.7	18.3 ± 5.7	18.3 ± 5.7	18.3 ± 5.7

Means within the same column followed by * are significantly different at *p* ≤ 0.05, as determined by an ANOVA: LSD test.

## Discussion

Climate change is the most important factor influencing natural ecosystems and organisms (Howden et al., 2007; Jeong et al., 2018). The elevated CO_2_ concentration has attracted attention worldwide, and it is expected to drive wide spread increases in extreme heat events this century (Skinner et al., 2018). Organisms are more sensitive to abrupt than to gradual change, and extreme climate events are considered to be the main instigators of evolution (Gutschick and Bassiri Rad, 2003; Combes, 2008), thus climate change will result in a series of chain reactions, affecting organisms at all trophic levels (Rouault et al., 2006). On the other hand, extreme climatic events can enhance invasion processes, from initial introduction through to establishment and spread (Walther et al., 2009; Diez et al., 2012). Thus, in this study, we focused on the effect of elevated CO_2_ and heat waves on common ragweed secondary metabolism change and on the performance of the common ragweed-specialist herbivorous insect *O. communa* through metabolomics and a two-sex life table, respectively. Subsequently, we will demonstrate the interaction of common ragweed–leaf beetle under a climate change context and providing guidance of common ragweed biocontrol in the future.

Studies have reported that elevated CO_2_ has a beneficial impact on many aspects of C3 plants, such as growth rate, biomass, photosynthetic rate (Wong, 1979; Rogers et al., 1983; Specht et al., 2014; Obermeier et al., 2017), as well as the variation of carbon-based plant secondary metabolites (Bryant et al., 1983; Herms and Mattson, 1992). Plant secondary metabolites play important roles in various aspects of plant biology, including regulation of growth (Mikkelsen et al., 2015), suppression of competing plants (Inderjit, 1996), and protection of plants from adverse environments (Veteli et al., 2003; Zhao et al., 2016). Previous studies have reported that high levels of CO_2_ induce plants to increase their accumulation of carbon-based secondary compounds, while gentle warming decreases the production of secondary compounds, counteracting the defensive effects of plants induced by high CO_2_ concentrations (Lincoln et al., 1993; Veteli et al., 2002). In the present study, however, elevated CO_2_ alone, heat wave alone, and a combination of elevated CO_2_ and heat wave remarkably enhanced the concentrations of total phenolics and condensed tannins in common ragweed. In addition, unlike gentle warming, which counteracts the plant defensive response induced by high CO_2_, the heat wave significantly intensified the defensive response of plants, resulting in more phenolics and condensed tannins. This suggests that there is a significant synergetic relationship between elevated atmospheric CO_2_ concentration and heat waves, which stimulate more intense defensive responses in plants. Thus increased secondary compound accumulation in plants will probably happen under future climate change conditions. Metabolomic profile results showed that the concentrations of 163 metabolites changed significantly in the leaves of common ragweed after combined treatment with elevated CO_2_ and heat waves (Figure 5; Supplementary Figure 5). Moreover, the content of N-(p-coumaroyl) serotonin in common ragweed leaves under the elevated CO_2_ and heat waves treatment was 2264.4-fold higher than control treatment. This large-scale change in secondary metabolites demonstrated that common ragweed had a strong ability to activate its defense system and adjust the secondary metabolite pathways to resist the unfavorable environment, which will facilitate common ragweed to better adapt to future climate change and develop its invasion potential.

Another important function of plant secondary metabolites is defense against herbivores (Rausher, 2001; Jia et al., 2014). Previous studies have reported that secondary metabolites like alkaloids, flavonol glycosides, gossypol, and tannins are toxic to insects, resulting in slow growth of insects, inhibition of molting, and decreased fecundity (Traw et al., 1996; Lindroth et al., 1997; Canals et al., 2005; Stipanovic et al., 2006). Thus, climate change likely would also influence insect performance and affect plant-herbivore interactions through changes in the levels of secondary metabolites in plants. We found that elevated CO_2_ alone, heat wave alone, and a combination of elevated CO_2_ and heat wave increased *O. communa* developmental period (larva and pupa stages) but decreased their survival rate, adult longevity, and fecundity. Compared with the control treatment, the intrinsic rate of increase and net reproduction rate significantly decreased but generation time significantly increased in the other three treatments. This signifies that feeding on common ragweed grown under elevated CO_2_ and heat wave conditions had a profound impact on the development and reproduction of *O. communa* populations. Besides enzyme activity results showed that the activities of six important enzymes in beetles (male and female) fed on common ragweed treated with elevated CO_2_ were significantly upregulated, and beetles fed on common ragweed treated with elevated CO_2_ and heat wave had highest EST enzyme activities. This is because elevated CO_2_ and heat wave increased the concentration of secondary metabolites in common ragweed (e.g., flavonoid and tannins; Figures 1, 2, 5). While detoxification and digestion process of insect require many nutrients and energy (Schoonhoven and Meerman, 1978). Therefore, when *O. communa* responds to the defensive reaction of common ragweed induced by high concentrations of CO_2_ and heat waves, it needs to allocate more energy for detoxification and digestion, which will prolong the development time and reduce spawning. The above results—including the development period, fecundity, survival rate, life table parameter, and enzyme activity of *O. communa—*show that heat waves strengthen the defense effect of common ragweed against herbivores through changes in the levels of secondary metabolites under elevated CO_2_ conditions. In the next step, we selected eight significantly upregulated substances for a bioassay test. The results showed that the mortality rates of *O. communa* (immature stage) under six substances were significantly higher than those in the control group, demonstrating that the combination of elevated CO_2_ and heat wave-induced secondary metabolites was unfavorable for the development of *O. communa*. Consistent with our results, Jamieson et al. (2015) reported that warming induced higher levels of condensed tannin and phenolic glycoside in *Populus tremuloides* and markedly increased larval development times and decreased larval weight and food conversion efficiency. Thus, climate change induced changes in phytochemicals leading to corresponding host species-specific changes in insect performance (Jamieson et al., 2015). Above all, it is predicted that the increase in secondary metabolites in common ragweed caused by heat wave and elevated CO_2_ will bring great challenges to the biological control of common ragweed in the future.

As a worldwide allergenic and invasive weed, common ragweed has brought great trouble to agriculture and human health. Therefore, prediction of the performance of common ragweed under a climatic change trend is beneficial to prevent its distribution and reduce its harm to human health. It is indisputable that the concentration of CO_2_ in the atmosphere will rise in the future and will increase the global temperature and drive the frequency and intensity of heat waves (Skinner et al., 2018). Previous studies have demonstrated that elevated CO_2_ and warming will boost common ragweed biomass, reproduction, pollen production, and allergenic ability (Rogers et al., 2006; Stinson et al., 2006; Stinson and Bazzaz, 2006; Sun et al., 2020). Sun et al. (2020) also demonstrated that common ragweed populations can rapidly evolve in response to climate change within a single generation, which will aggravate the impact of its distribution and damage in the future. Moreover, our results demonstrated that elevated CO_2_ and more extreme heat events can help common ragweed improve photosynthesis, increase total leaf area, and increase growth (Supplementary Figures 1–3), which will undoubtedly increase the competitive invasion ability of this species in the future. On the other hand, elevated CO_2_ and heat wave-induced accumulation of secondary metabolites in common ragweed had an unfavorable impact on the development of *O. communa*. Under elevated CO_2_ conditions, heat waves can promote common ragweed’s ability to adapt to climate change and defense against herbivores, making it more difficult to prevent and control common ragweed in the future.

## Data availability statement

The original contributions presented in the study are included in the article/Supplementary material, further inquiries can be directed to the corresponding author.

## Author contributions

ZZ designed the study and revised the manuscript. Together, ZhenyT and CM performed the research and wrote the manuscript. CZ performed the data analyses. YZ, XG, and ZhenqT participated in sample collection and data sorting. HC and JG provided guidance during the data analysis. All authors contributed to the article and approved the submitted version.

## Funding

This work was supported by the National Natural Science Foundation of China (32172494 and 31672089).

## Conflict of interest

The authors declare that the research was conducted in the absence of any commercial or financial relationships that could be construed as a potential conflict of interest.

## Publisher’s note

All claims expressed in this article are solely those of the authors and do not necessarily represent those of their affiliated organizations, or those of the publisher, the editors and the reviewers. Any product that may be evaluated in this article, or claim that may be made by its manufacturer, is not guaranteed or endorsed by the publisher.

## Supplementary material

Supplementary Figure 1 Leaf photosynthesis intensity (± SE) of *Ambrosia artemisiifolia* under different stress conditions. Data represented by columns bearing the same letters were not significantly different (LSD, *p* = 0.05). AN: net photosynthesis; a, b, c, d, and e represent the 1st, 2nd, 3rd, 4th, and 5th measurements, respectively. AT denotes the ambient temperature condition; HW denotes the heat wave condition. EC and AC represent elevated atmosphere CO2 concentration and ambient atmosphere CO2 concentration, respectively.

Supplementary Figure 2 Total leaf area (±SE) of *Ambrosia artemisiifolia* under different stress conditions. Data represented by columns bearing the same letters were not significantly different (LSD, p = 0.05). AT denotes the ambient temperature condition; HW denotes the heat wave condition. EC and AC represent elevated atmosphere CO2 concentration and ambient atmosphere CO2 concentration, respectively.

Supplementary Figure 3 Plant height (±SE) of *Ambrosia artemisiifolia* under different stress conditions. Data represented by columns bearing the same letters were not significantly different (LSD, p = 0.05). a, b, c, d, and e represent the 1st, 2nd, 3rd, 4th, and 5th measurements, respectively. AT denotes the ambient temperature condition; HW denotes the heat wave condition. EC and AC represent elevated atmospheric CO2 concentration and ambient atmosphere CO2 concentration, respectively.

Supplementary Figure 4 KEGG analysis of different metabolites between leaves of plants under AC + AT and EC + HW conditions.

Supplementary Figure 5 Heatmap of different metabolites between leaves of plants under AC + AT and EC + HW conditions. CK:AC + AT; AT denotes the ambient temperature condition; HW denotes the heat wave condition. EC and AC represent elevated atmospheric CO2 concentration and ambient atmosphere CO2 concentration, respectively.

## Abbreviations

ANOVA: Analysis of variance
BapNA: Benzoyl-L-arg-p-nitroanilide
FC: Fold change
KEGG: Kyoto encyclopedia of genes and genomes
MRM: Multiple reaction monitoring
PCA: Principal component analysis
PPFD: Photosynthetic photon flux density
QC: Quality control
RH: Relative humidity

## References

[ref1] Aebi H. (1984). Catalase in vitro. Meth. Enzymol. 105, 121–126. doi: 10.1016/s0076-6879(84)05016-3, PMID: 6727660

[ref001] Ainsworth E. A. Rogers A. (2007). The response of photosynthesis and stomatal conductance to rising [CO2]: mechanisms and environmental interactions. Plant Cell Environ. 303, 258–270., PMID: 17263773 10.1111/j.1365-3040.2007.01641.x

[ref2] Bauweraerts I. Ameye M. Wertin T. M. McGuire M. A. Teskey R. O. Steppe K. (2014). Water availability is the decisive factor for the growth of two tree species in the occurrence of consecutive heat waves. Agric. For. Meteorol. 189-190, 19–29. doi: 10.1016/j.agrformet.2014.01.001

[ref4] Bryant J. P. Chapin F. S. Klein D. R. (1983). Carbon/nutrient balance of boreal plants in relation to vertebrate herbivory. Oikos 40, 357–368. doi: 10.2307/3544308

[ref5] Buse A. Good J. E. G. Dury S. Perrins C. M. (1998). Effects of elevated temperature and carbon dioxide on the nutritional quality of leaves of oak (*Quercus robur* L.) as food for the winter moth (Operophterabrumata L.). Funct. Ecol. 12, 742–749. doi: 10.1046/j.1365-2435.1998.00243.x

[ref6] Canals D. Irurre-Santilari J. Casas J. (2005). The first cytochrome P450 in ferns. Evidence for its involvement in phytoecdysteroid biosynthesis in *Polypodium vulgare*. FEBS J. 272, 4817–4825. doi: 10.1111/j.1742-4658.2005.04897.x, PMID: 16156800

[ref7] Chen F. Feng G. Megha N. P. (2005). Impact of elevated CO_2_ on tri-trophic interaction of *Gossypium Hirsutum*, aphis Gossypii, and leis Axyridis. Physiol. Ecol. 34, 37–46. doi: 10.1603/0046-225X-34.1.37

[ref8] Chen W. Gong L. Guo Z. Wang W. Zhang H. Liu X.. (2013). A novel integrated method for large-scale detection, identification, and quantification of widely targeted metabolites: application in the study of rice metabolomics. Mol. Plant 6, 1769–1780. doi: 10.1093/mp/sst080, PMID: 23702596

[ref002] Chi H. (1988). Life-table analysis incorporating both sexes and variable development rates among individuals. Environ. Entomol. 17, 26–34.

[ref003] Chi H. Smith C. L. (2014). Age-stage, two-sex life table: Theory, data analysis, and application.

[ref10] Ciais P. Reichstein M. Viovy N. Granier A. Ogée J. Allard V.. (2005). Europe-wide reduction in primary productivity caused by the heat and drought in 2003. Nature 437, 529–533. doi: 10.1038/nature03972, PMID: 16177786

[ref11] Combes C. (2008). The role of extreme events in evolution. Compt. Rendus Geosci. 340, 591–594. doi: 10.1016/j.crte.2008.01.003, PMID: 35488861

[ref004] Cowbrough M. J. Brown R. B. Tardif F. J. (2003). Impact of common ragweed (Ambrosia artemisiifolia) aggregation on economic thresholds in soybean. Weed Sci. 51, 947–954. doi: 10.1614/02-036

[ref12] Curtis P. S. Wang X. (1998). A meta-analysis of elevated CO2 effects on woody plant mass, form, and physiology. Oecologia 113, 299–313. doi: 10.1007/s004420050381, PMID: 28307814

[ref13] Diez J. M. D’ Antonio C. M. Dukes J. S. Grosholz E. D. Olden J. D. Sorte C. J. B.. (2012). Will extreme climatic events facilitate biological invasions? Front. Ecol. Environ. 10, 249–257. doi: 10.1890/110137

[ref14] Drake B. L. Hanson D. T. Lowrey T. K. Sharp Z. D. (2017). The carbon fertilization effect over a century of anthropogenic CO_2_ emissions: higher intracellular CO_2_ and more drought resistance among invasive and native grass species contrasts with increased water use efficiency for woody plants in the US southwest. Glob. Chang. Biol. 23, 782–792. doi: 10.1111/gcb.13449, PMID: 27483457

[ref15] El Kelish A. E. Zhao F. Heller W. Durner J. Winkler J. B. Behrendt H.. (2014). Ragweed (*Ambrosia artemisiifolia*) pollen allergenicity: super SAGE transcriptomic analysis upon elevated CO2 and drought stress. BMC Plant Biol. 14:176. doi: 10.1186/1471-2229-14-176, PMID: 24972689 PMC4084800

[ref16] Ellman G. L. Courtney K. D. Andres V. Featherstone R. M. (1961). A new and rapid colorimetric determination of acetylcholinesterase activity. Biochem. Pharmacol. 7, 88–95. doi: 10.1016/0006-2952(61)90145-9, PMID: 13726518

[ref17] Fajer E. D. (1989). The effects of enriched CO_2_ atmospheres on plant-insect herbivore interactions: growth responses of larvae of the specialist butterfly, *Junonia Coenia* (Lepidoptera: Nymphalidae). Oecologia 81, 514–520. doi: 10.1007/BF00378962, PMID: 28312647

[ref18] García-Herrera R. Díaz J. Trigo R. M. Luterbacher J. Fischer E. M. (2010). A review of the European summer heat wave of 2003. Crit. Rev. Environ. Sci. Technol. 40, 267–306. doi: 10.1080/10643380802238137, PMID: 16773557

[ref20] Gutschick V. P. Bassiri Rad H. (2003). Extreme events as shaping physiology, ecology, and evolution of plants: toward a unified definition and evaluation of their consequences. New Phytol. 160, 21–42. doi: 10.1046/j.1469-8137.2003.00866.x, PMID: 33873544

[ref22] Herms D. A. Mattson W. J. (1992). The dilemma of plants: to grow or defend. Q. Rev. Biol. 67, 283–335. doi: 10.1086/417659, PMID: 35796366

[ref24] Howden S. M. Soussana J. F. Tubiello F. N. Chhetri N. Dunlop M. Meinke H. (2007). Adapting agriculture to climate change. Proc. Natl. Acad. Sci. U. S. A. 104, 19691–19696. doi: 10.1073/pnas.0701890104, PMID: 18077402 PMC2148359

[ref25] Ibrahim M. H. Jaafar H. Z. E. (2012). Impact of elevated carbon dioxide on primary, secondary metabolites and antioxidant responses of *Eleaisguineensis*Jacq. (oil palm) seedlings. Molecules 17, 5195–5211. doi: 10.3390/molecules17055195, PMID: 22628041 PMC6268660

[ref26] Inderjit (1996). Plant phenolics in allelopathy. Bot. Rev. 62, 186–202. doi: 10.1007/BF02857921, PMID: 35567121

[ref005] IPCC (2013). Climate Change 2013: The Physical Science Basis. Contribution of Working Group I to the Fifth Assessment Report of the Intergovernmental Panel on Climate Change. Cambridge University Press, Cambridge, United Kingdom and New York, NY, USA, pp 1535.

[ref27] IPCC (2021). Climate Change 2021: The Physical Science Basis. Contribution of Working Group I to the Sixth Assessment Report of the Intergovernmental Panel on Climate Change. eds. Masson-Delmotte, V., P. Zhai, A. Pirani, S. L. Connors, C. Péan, S. Berger, N. Caud, Y. Chen, L. Goldfarb, M. I. Gomis, *M. Huang*, K. Leitzell, E. Lonnoy, J. B. R. Matthews, T. K. Maycock, T. Waterfield, O. Yelekçi, R. Yu, and B. Zhou. Cambridge University Press, Cambridge, United Kingdom and New York, NY, USA, In press.

[ref28] Jamieson M. A. Schwartzberg E. G. Raffa K. F. Reich P. B. Lindroth R. L. (2015). Experimental climate warming alters aspen and birch phytochemistry and performance traits for an outbreak insect herbivore. Glob. Chang. Biol. 21, 2698–2710. doi: 10.1111/gcb.12842, PMID: 25538021

[ref29] Jeong H.-M. Kim H.-R. Hong S. You Y.-H. (2018). Effects of elevated CO2 concentration and increased temperature on leaf quality responses of rare and endangered plants. J. Ecol. Environ. 42:1. doi: 10.1186/s41610-017-0061-0

[ref30] Jia X. Wang W. K. Chen Z. H. He Y. H. Liu J. X. (2014). Concentrations of secondary metabolites in tissues and root exudates of wheat seedlings changed under elevated atmospheric CO_2_ and cadmium-contaminated soils. Environ. Exp. Bot. 107, 134–143. doi: 10.1016/j.envexpbot.2014.06.005

[ref31] Johns C. V. Beaumont L. J. Hughes L. (2003). Effects of elevated CO2 and temperature on development and consumption rates of *Octotomachampioni* and *O. scabripennis* feeding on *Lantana camara*. Entomol. Exp. Appl. 108, 169–178. doi: 10.1046/j.1570-7458.2003.00076.x

[ref32] Johns C. V. Hughes L. (2002). Interactive effects of elevated CO2 and temperature on the leaf-miner *Dialecticascalariella* Zeller (Lepidoptera: Gracillariidae) in Paterson’s curse*, Echium plantagineum* (Boraginaceae). Glob. Chang. Biol. 8, 142–152. doi: 10.1046/j.1365-2486.2002.00462.x

[ref006] Kandil M. A. Abdallah I. S. Abou-yousef H. M. Abdallah N. A. Fouad E. A. (2017). Mechanism of resistance to pirimicarb in the cowpea aphid Aphis craccivora. Crop Prot. 94, 173–177.

[ref33] Kanerva S. Smolander A. (2008). How do coniferous needle tannins influence C and N transformations in birch humus layer? Eur. J. Soil Biol. 44, 1–9. doi: 10.1016/j.ejsobi.2007.08.001

[ref34] Karowe D. N. (2007). Are legume-feeding herbivores buffered against direct effects of elevated carbon dioxide on host plants? A test with the sulfur butterfly, *Coliasphilodice*. Glob. Chang. Biol. 13, 2045–2051. doi: 10.1111/j.1365-2486.2007.01422.x

[ref007] Körner C. (2000). Biosphere responses to CO_2_ enrichment. Ecol. Appl. 10, 1590–1619.

[ref35] Lee Y. H. Sang W. G. Baek J. K. Kim J. H. Shin P. Seo M. C.. (2020). The effect of concurrent elevation in CO2 and temperature on the growth, photosynthesis, and yield of potato crops. PLoS One 15:e0241081. doi: 10.1371/journal.pone.0241081, PMID: 33085713 PMC7577495

[ref36] Lincoln D. E. Fajer E. D. Johson R. H. (1993). Plant–insect herbivore interactions in elevated CO(2) environments. Trends Ecol. Evol. 8, 64–68. doi: 10.1016/0169-5347(93)90161-H, PMID: 21236109

[ref37] Lindroth R. L. Roth S. Kruger E. L. Volin J. C. Koss P. A. (1997). CO_2_-mediated changes in aspen chemistry: effects on gypsy moth performance and susceptibility to virus. Glob. Chang. Biol. 3, 279–289. doi: 10.1046/j.1365-2486.1997.00077.x

[ref39] Marklund S. Marklund G. (1974). Involvement of the superoxide anion radical in the autoxidation of pyrogallol and a convenient assay for superoxide dismutase. Eur. J. Biochem. 47, 469–474. doi: 10.1111/j.1432-1033.1974.tb03714.x, PMID: 4215654

[ref40] Mikkelsen B. L. Olsen C. E. Lyngkjær M. F. (2015). Accumulation of secondary metabolites in healthy and diseased barley, grown under future climate levels of CO_2_, ozone and temperature. Phytochemistry 118, 162–173. doi: 10.1016/j.phytochem.2015.07.007, PMID: 26343414

[ref41] Obermeier W. A. Lehnert L. W. Kammann C. I. Muller C. Grunhage L. Luterbacher J.. (2017). Reduced CO2 fertilization effect in temperature C3 grasslands under more extreme weather conditions. Nat. Clim. Chang. 7, 137–141. doi: 10.1038/nclimate3191

[ref42] Poorter H. van Berkel Y. Baxter B. Hertog J. D. Dijkstra P. Gifford R. M.. (1997). The effect of elevated CO2 on the chemical composition and construction costs of leaves. Plant Cell Environ. 20, 472–482. doi: 10.1046/j.1365-3040.1997.d01-84.x

[ref43] Rausher M. D. (2001). Co-evolution and plant resistance to natural enemies. Nature 411, 857–864. doi: 10.1038/35081193, PMID: 11459070

[ref44] Rogers H. H. Thomas J. F. Bingham G. E. (1983). Response of agronomic and forest species to elevated atmospheric carbon dioxide. Science 220, 428–429. doi: 10.1126/science.220.4595.428, PMID: 17831416

[ref45] Rogers C. A. Wayne P. M. Macklin E. A. Muilenberg M. L. Wagner C. J. Epstein P. R.. (2006). Interaction of the onset of spring and elevated atmospheric CO2 on ragweed (*Ambrosia artemisiifolia* L.) pollen production. Environ. Health Perspect. 114, 865–869. doi: 10.1289/ehp.8549, PMID: 16759986 PMC1480488

[ref46] Rouault G. Candau J. N. Lieutier F. Nageleisen L. M. Martin J. C. Warzée N. (2006). Effects of drought and heat on forest insect populations in relation to the 2003 drought in Western Europe. Ann. For. Sci. 63, 613–624. doi: 10.1051/forest:2006044

[ref47] Schädler M. Roeder M. Brandl R. Matthies D. (2007). Interacting effects of elevated CO_2_, nutrient availability and plant species on a generalist invertebrate herbivore. Glob. Chang. Biol. 13, 1005–1015. doi: 10.1111/j.1365-2486.2007.01319.x

[ref48] Schaffner U. Steinbach S. Sun Y. Skjøth C. A. de Weger L. A. Lommen S. T.. (2020). Biological weed control to relieve millions from Ambrosia allergies in Europe. Nat. Commun. 11:1745. doi: 10.1038/s41467-020-15586-1, PMID: 32317698 PMC7174423

[ref49] Schoonhoven L. M. Meerman J. (1978). Metabolic cost of changes in diet and neutralization of allelochemics. Entomol. Exp. Appl. 24, 689–693. doi: 10.1111/j.1570-7458.1978.tb02833.x

[ref50] Seo E. Lee M. I. Jeong J. H. Koster R. D. Schubert S. D. Kim H.-M.. (2019). Impact of soil moisture initialization on boreal summer subseasonal forecasts: mid-latitude surface air temperature and heat wave events. Clim. Dyn. 52, 1695–1709. doi: 10.1007/s00382-018-4221-4

[ref51] Skinner C. B. Poulsen C. J. Mankin J. S. (2018). Amplification of heat extremes by plant CO_2_ physiological forcing. Nat. Commun. 9:1094. doi: 10.1038/s41467-018-03472-w, PMID: 29545570 PMC5854667

[ref52] Specht J. E. Diers B. W. Nelson R. L. de Toledo J. F. F. Torrion J. A. Grassini P. (2014). “Soybean,” in Yield Gains Majors. Field Crops. eds. Smith S. Diers B. Specht J. Carver B. (Madison (WI): American Society of Agronomy), 311–355.

[ref53] Stiling P. Tatiana C. (2007). How does elevated carbon dioxide (CO_2_) affect plant – herbivore interactions? A field experiment and Meta-analysis of CO_2_-mediated changes on plant chemistry and herbivore performance. Glob. Chang. Biol. 13, 1823–1842. doi: 10.1111/j.1365-2486.2007.01392.x

[ref54] Stinson K. A. Bazzaz F. A. (2006). CO(2)-enrichment reduces reproductive dominance in competing stands of *Ambrosia artemisiifolia* (common ragweed). Oecologia 147, 155–163. doi: 10.1007/s00442-005-0250-x, PMID: 16163552

[ref55] Stinson K. A. Tran J. H. Petzold J. L. Bazzaz F. A. (2006). Architectural and physiological mechanisms of reduced size inequality in CO_2_-enriched stands of common ragweed (*Ambrosia artemisiifolia*). Glob. Chang. Biol. 12, 1680–1689. doi: 10.1111/j.1365-2486.2006.01229.x

[ref56] Stipanovic R. D. Lopez J. D. Dowd M. K. Puckhaber L. S. Duke S. E. (2006). Effect of racemic and (+)-and (−)-gossypol on the survival and development of *Helicoverpa zea* larvae. J. Chem. Ecol. 32, 959–968. doi: 10.1007/s10886-006-9052-9, PMID: 16739016

[ref57] Sun Y. Bossdorf O. Grados R. D. Liao Z. Müller-Schärer H. (2020). Rapid genomic and phenotypic change in response to climate warming in a widespread plant invader. Glob. Chang. Biol. 26, 6511–6522. doi: 10.1111/gcb.15291, PMID: 32702177

[ref59] Sun Y. C. Guo H. J. Yuan E. L. Ge F. (2018). Elevated CO_2_ increases R gene-dependent resistance of *Medicago truncatula* against the pea aphid by up-regulating a heat shock gene. New Phytol. 217, 1696–1711. doi: 10.1111/nph.14892, PMID: 29154460

[ref61] Tong H. Cai L. Zhou G. Yuan T. Zhang W. Tian R.. (2017). Temperature shapes coral-algal symbiosis in the South China Sea. Sci. Rep. 7:40118. doi: 10.1038/srep40118, PMID: 28084322 PMC5234030

[ref63] Traw M. B. Lindroth R. L. Bazzaz F. A. (1996). Decline in gypsy moth (*Lymantria dispar*) performance in an elevated CO2 atmosphere depends upon host plant species. Oecologia 108, 113–120. doi: 10.1007/BF00333222, PMID: 28307741

[ref64] Tripathee R. (2008). Effect of CO2 on the Response of C and N Relations to a Heat Wave in Sun-Flower and Corn. Master's thesis. University of Toledo. Available at: http://rave.ohiolink.edu/etdc/view?acc_num=toledo1218558510 (Accessed July 15, 2022).

[ref65] Veteli T. O. Kuokkanen K. Julkunen-Tiitto R. Roininen H. Tahvanainen J. (2002). Effects of elevated CO_2_ and temperature on plant growth and herbivore defensive chemistry. Glob. Chang. Biol. 8, 1240–1252. doi: 10.1046/j.1365-2486.2002.00553.x, PMID: 29762698

[ref66] Veteli T. O. Mattson W. J. Niemelä P. Julkunen-Tiitto R. Kellomäki S. Kuokkanen K.. (2007). Do elevated temperature and CO_2_ generally have counteracting effects on phenolic phytochemistry of boreal trees? J. Chem. Ecol. 33, 287–296. doi: 10.1007/s10886-006-9235-4, PMID: 17216360

[ref67] Veteli T. O. Tegelberg R. Pusenius J. Sipura M. Julkunen-Tiitto R. Aphalo P. J.. (2003). Interactions between willows and insect herbivores under enhanced ultraviolet-B radiation. Oecologia 137, 312–320. doi: 10.1007/s00442-003-1298-0, PMID: 12908105

[ref68] Vinagre C. Mendonça V. Cereja R. Abreu-Afonso F. Dias M. Mizrahi D.. (2018). Ecological traps in shallow coastal waters-potential effect of heat-waves in tropical and temperate organisms. PLoS One 13:e0192700. doi: 10.1371/journal.pone.0192700, PMID: 29420657 PMC5805332

[ref69] Visweshwar R. Sharma H. C. Akbar S. M. D. Sreeramulu K. (2015). Elimination of gut microbes with antibiotics confers resistance to *Bacillus thuringiensis* toxin proteins in Helicoverpaarmigera (Hubner). Appl. Biochem. Biotechnol. 177, 1621–1637. doi: 10.1007/s12010-015-1841-6, PMID: 26384494

[ref71] Walther G. Roques A. Hulme P. E. Sykes M. T. Pysek P. Kuhn I.. (2009). Alien species in a warmer world: risks and opportunities. Trends Ecol. Evol. 24, 686–693. doi: 10.1016/j.tree.2009.06.008, PMID: 19712994

[ref72] Wayne P. Foster S. Connolly J. Bazzaz F. Epstein P. (2002). Production of allergenic pollen by ragweed (*Ambrosia artemisiifolia* L.) is increased in CO2-enriched atmospheres. Ann. Allergy Asthma Immunol. 88, 279–282. doi: 10.1016/S1081-1206(10)62009-1, PMID: 11926621

[ref73] Williams R. S. Norby R. J. Lincoln D. E. (2000). Effects of elevated CO2 and temperature-grown red and sugar maple on gypsy moth performance. Glob. Chang. Biol. 6, 685–695. doi: 10.1046/j.1365-2486.2000.00343.x

[ref74] WMO Greenhouse Gas Bulletin (2018). GHG bulletin, 2018—The State of Greenhouse Gases in the Atmosphere Based on Global Observations through 2017—No. 14. World Meteorological Organization (WMO).

[ref75] Wong S. C. (1979). Elevated atmospheric partial pressure of CO2 and plant growth: I. interactions of nitrogen nutrition and photosynthetic capacity in C3 and C4 plants. Oecologia 44, 68–74. doi: 10.1007/BF00346400, PMID: 28310466

[ref77] Zhao Y. H. Jia X. Wang W. K. Liu T. Huang S. P. Yang M. Y. (2016). Growth under elevated air temperature altered secondary metabolites in *Robinia pseudoacacia* L. seedlings in cd- and Pb-contaminated soils. Sci.Total Environ. 565, 586–594. doi: 10.1016/j.scitotenv.2016.05.058 27203519

[ref78] Zhou Z. S. Guo J. Y. Chen H. S. Wan F. H. (2010). Effects of temperature on survival, development, longevity, and fecundity of *Ophraella communa* (Coleoptera: Chrysomelidae), a potential biological control agent against *Ambrosia artemisiifolia* (Asterales: Asteraceae). Environ. Entomol. 39, 1021–1027. doi: 10.1603/EN09176, PMID: 20550818

[ref79] Ziska L. H. Caulfield F. A. (2000). Rising CO2 and pollen production of common ragweed (*Ambrosia artemisiifolia* L.), a known allergy-inducing species: implications for public health. Funct. Plant Biol. 27, 893–898. doi: 10.1071/PP00032

[ref80] Zvereva E. L. Kozlov M. V. (2006). Consequences of simultaneous elevation of carbon dioxide and temperature for plant–herbivore interactions: a metaanalysis. Glob. Chang. Biol. 12, 27–41. doi: 10.1111/j.1365-2486.2005.01086.x

