# Supplementary figures and images for: Heat wave event facilitates defensive responses in invasive C3 plant Ambrosia artemisiifolia L. under elevated CO2 concentration to the detriment of Ophraella communa
Source: Front Plant Sci. 2022 Jul 27;13:907764. doi: 10.3389/fpls.2022.907764 (PMC9363847; doi:10.3389/fpls.2022.907764)

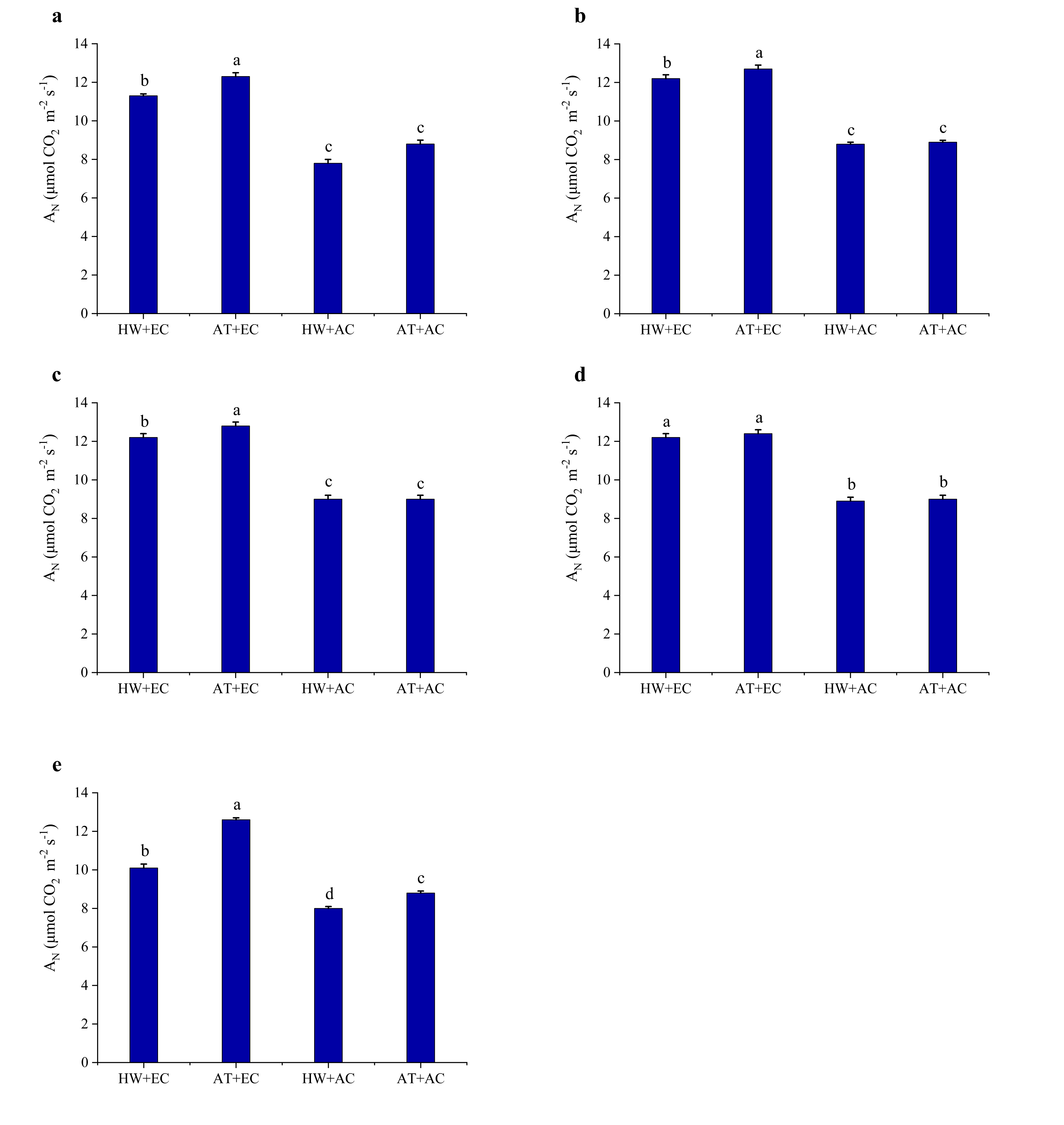

Supplement: Supplementary Figure 1 — Leaf photosynthesis intensity (± SE) of Ambrosia artemisiifolia under different stress conditions. Data represented by columns bearing the same letters were not significantly different (LSD, p = 0.05). AN: net photosynthesis; a, b, c, d, and e represent the 1st, 2nd, 3rd, 4th, and 5th measurements, respectively. AT denotes the ambient temperature condition; HW denotes the heat wave condition. EC and AC represent elevated atmosphere CO2 concentration and ambient atmosphere CO2 concentration, respectively. [file Image_1.TIF]

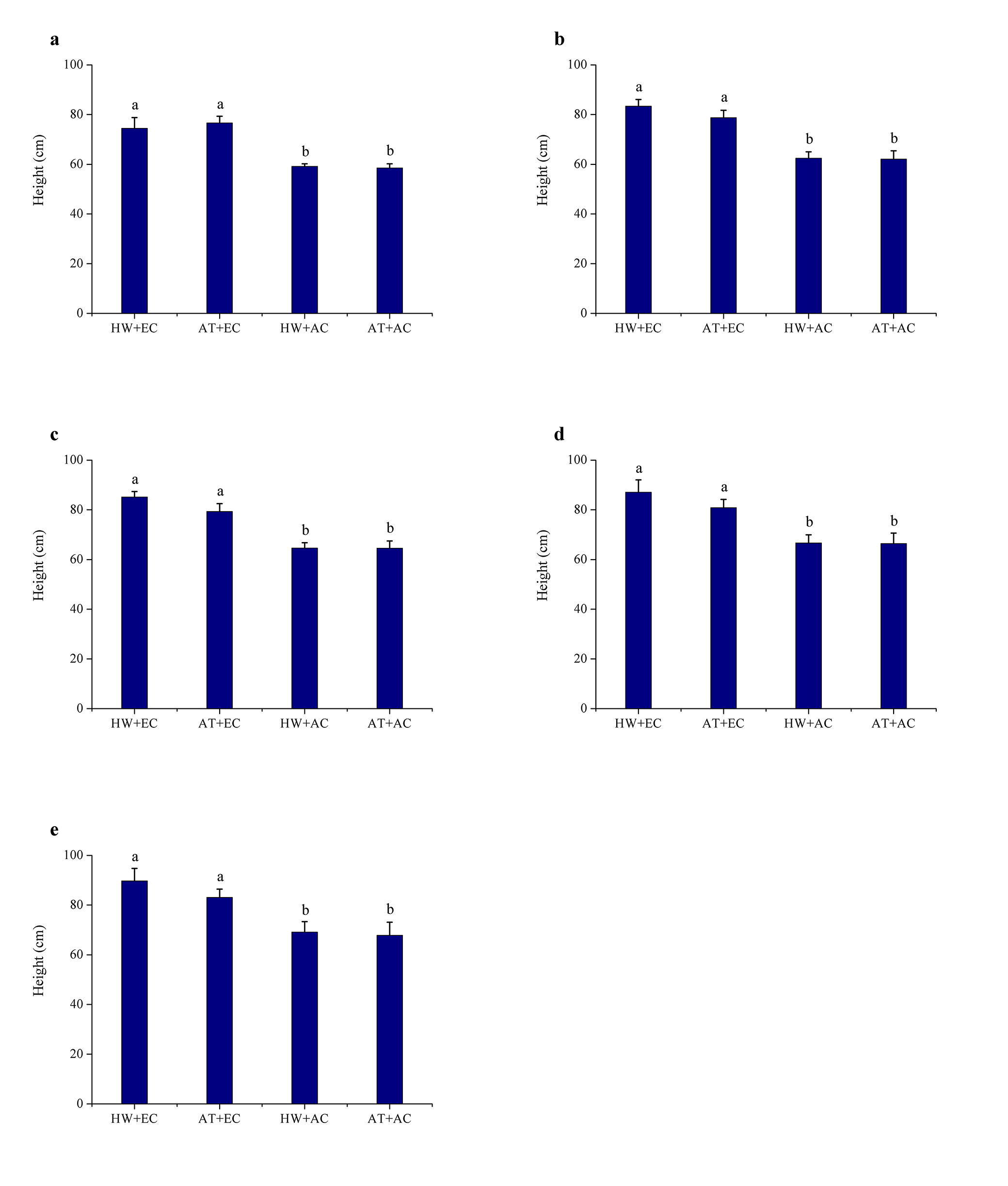

Supplement: Supplementary Figure 3 — Plant height (±SE) of Ambrosia artemisiifolia under different stress conditions. Data represented by columns bearing the same letters were not significantly different (LSD, p = 0.05). a, b, c, d, and e represent the 1st, 2nd, 3rd, 4th, and 5th measurements, respectively. AT denotes the ambient temperature condition; HW denotes the heat wave condition. EC and AC represent elevated atmospheric CO2 concentration and ambient atmosphere CO2 concentration, respectively. [file Image_3.TIF]

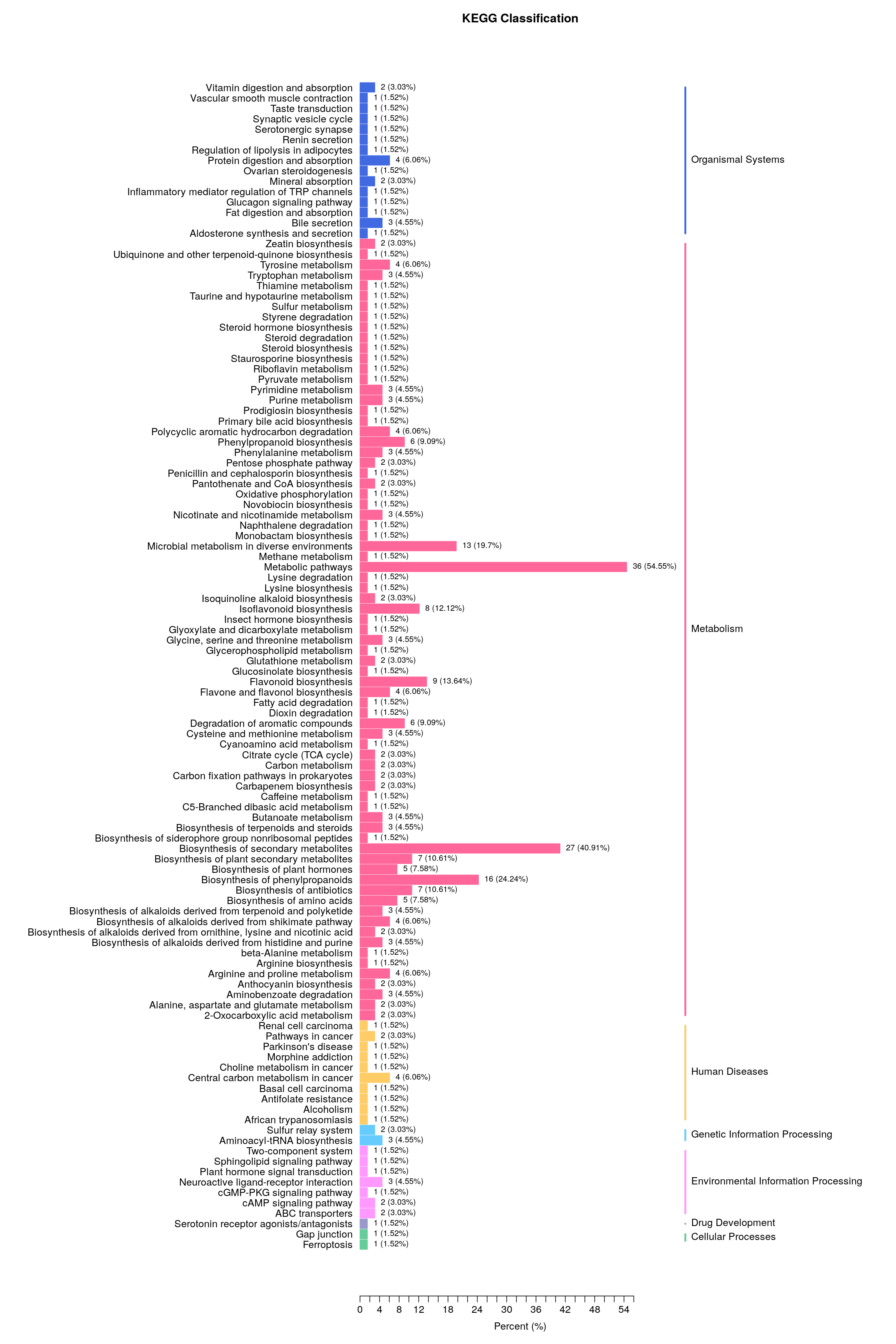

Supplement: Supplementary Figure 4 — KEGG analysis of different metabolites between leaves of plants under AC + AT and EC + HW conditions. [file Image_4.TIF]

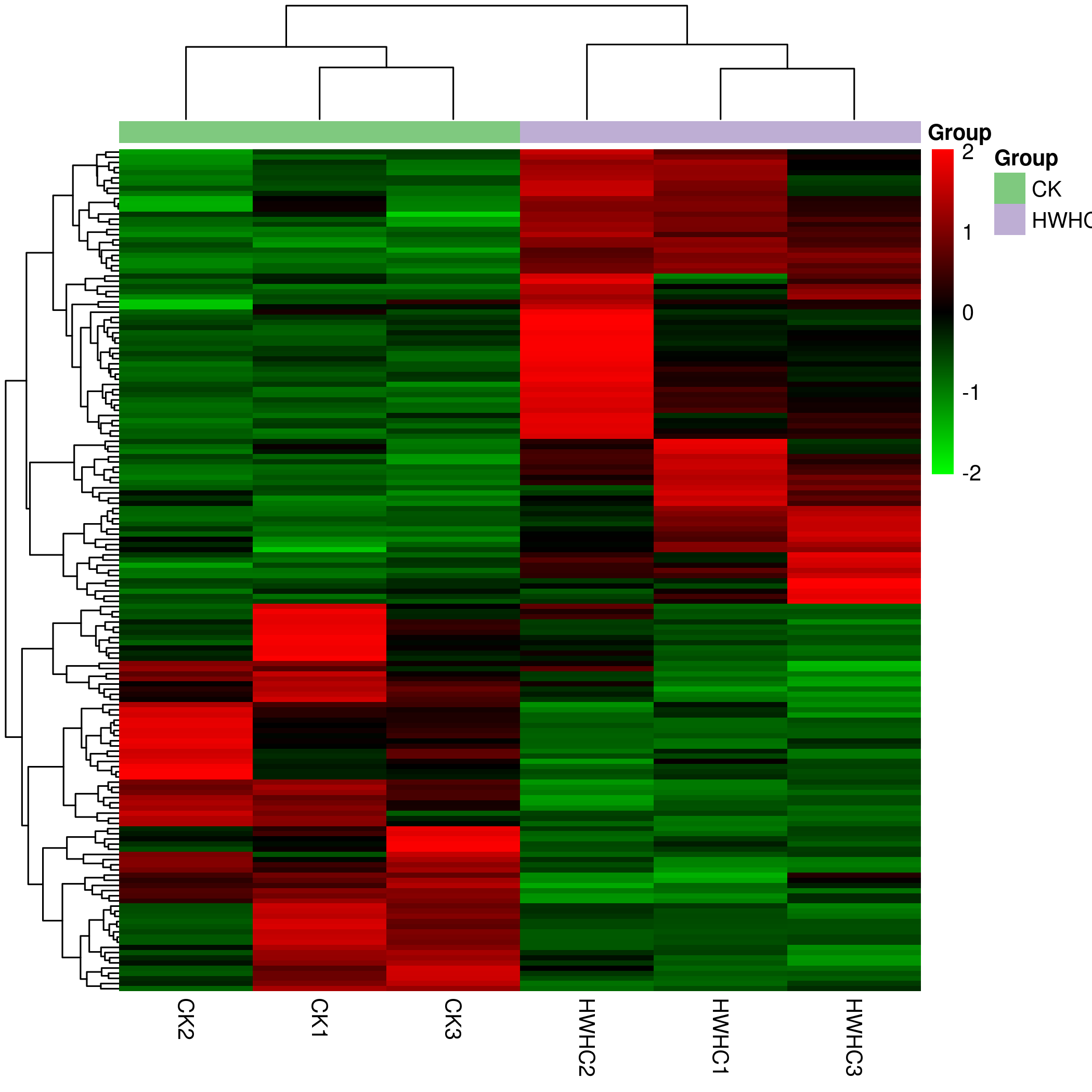

Supplement: Supplementary Figure 5 — Heatmap of different metabolites between leaves of plants under AC + AT and EC + HW conditions. CK:AC + AT; AT denotes the ambient temperature condition; HW denotes the heat wave condition. EC and AC represent elevated atmospheric CO2 concentration and ambient atmosphere CO2 concentration, respectively. [file Image_5.PNG]
